# Investigation of Phospholipase Cγ1 Interaction with SLP76 Using Molecular Modeling Methods for Identifying Novel Inhibitors

**DOI:** 10.3390/ijms20194721

**Published:** 2019-09-23

**Authors:** Neha Tripathi, Iyanar Vetrivel, Stéphane Téletchéa, Mickaël Jean, Patrick Legembre, Adèle D. Laurent

**Affiliations:** 1 CEISAM UMR CNRS 6230, UFR Sciences et Techniques, Université de Nantes, 44322 Nantes CEDEX 3, France; neha.tripathi@univ-nantes.fr (N.T.); iyanar.vetrivel@univ-nantes.fr (I.V.); 2 UFIP UMR CNRS 6286, UFR Sciences et Techniques, Université de Nantes, 44322 Nantes CEDEX 3, France; stephane.teletchea@univ-nantes.fr; 3 CLCC Eugène Marquis, Equipe Ligue Contre Le Cancer, 35042 Rennes, France; mickael.jean@univ-rennes1.fr (M.J.); patrick.legembre@inserm.fr (P.L.); 4 COSS INSERM UMR1242, Université Rennes 1, 35042 Rennes, France

**Keywords:** phospholipase C gamma 1, SLP76, virtual screening, pharmacophore mapping, molecular docking, molecular dynamics

## Abstract

The enzyme phospholipase C gamma 1 (PLCγ1) has been identified as a potential drug target of interest for various pathological conditions such as immune disorders, systemic lupus erythematosus, and cancers. Targeting its SH3 domain has been recognized as an efficient pharmacological approach for drug discovery against PLCγ1. Therefore, for the first time, a combination of various biophysical methods has been employed to shed light on the atomistic interactions between PLCγ1 and its known binding partners. Indeed, molecular modeling of PLCγ1 with SLP76 peptide and with previously reported inhibitors (ritonavir, anethole, daunorubicin, diflunisal, and rosiglitazone) facilitated the identification of the common critical residues (Gln805, Arg806, Asp808, Glu809, Asp825, Gly827, and Trp828) as well as the quantification of their interaction through binding energies calculations. These features are in agreement with previous experimental data. Such an in depth biophysical analysis of each complex provides an opportunity to identify new inhibitors through pharmacophore mapping, molecular docking and MD simulations. From such a systematic procedure, a total of seven compounds emerged as promising inhibitors, all characterized by a strong binding with PLCγ1 and a comparable or higher binding affinity to ritonavir (∆G_bind_ < −25 kcal/mol), one of the most potent inhibitor reported till now.

## 1. Introduction

Apoptosis [1] is genetically encoded to provide mechanisms related to organ formation, or to eliminate damaged cells. The mechanism is mediated by regulated interactions between various cellular components [2]. Under pathological conditions, such as cancers, autoimmune disorders and viral infections, dysregulated components might cause the decrease in cellular apoptosis [3]. Therefore, targeting the enzymes and receptors involved in regulation of cellular apoptosis has been established as an important therapeutic strategy for such pathological conditions [4,5,6,7,8,9,10,11,12]. Amongst the numerous regulators of apoptosis, the multifunctional phospholipase C (PLC) enzymes interact with target proteins to modulate the cellular apoptosis [13,14,15,16]. Indeed, PLCs are essential for regulation of several cellular processes as they catalyze the hydrolysis of phosphatidylinositol 4,5-bisphosphate (PIP2) into inositol 1,4,5-triphosphate (IP3) and diacylglycerol (DAG) using Ca^2+^ as cofactor [17]. In mammals, PLCγ isozyme is, particularly, involved in cell growth regulation [17,18,19,20], and is constituted of two isoforms (i.e., PLCγ1 and PLCγ2) [17]. The PLCγ1 isoform is constitutively expressed in all cells, whereas PLCγ2 is mainly expressed in immune cells [17]. Particularly, PLCγ1 has been identified to play an important role in the regulation of cell growth and cellular differentiation [17,19], by interacting with various macromolecular targets [17] such as epidermal growth factor, fibroblast growth factor, platelet derived growth factor, vascular endothelial growth factor and cluster of differentiation 95 (CD95) [16,21]. PLCγ1 is also known to be involved and to play an important role in cell invasion, metastasis and progression in cancers [13,15,22].

Structurally, PLCγ1 is a multidomain protein [14,21] (Figure S1), for which the catalytic site is present in a TIM barrel [19]. The catalytic activity of PLCγ1 is controlled by a conformational change in relative orientation of its various domains which, in turn, is governed by the phosphorylation of the Tyr783 residue [17]. The structural complexity of PLCγ1 contributes to the multitude of its biological targets. Particularly, the SH3 domain of PLCγ1 (PLCγ1-SH3) has been reported to contain the binding site for several target proteins, enriched in proline (PXXP motifs) [18]. Experimental studies ascertain the importance of the PLCγ1-SH3 in interactions with several proteins including autoimmune poly-endocrinopathy candidiasisectodermal dystrophy protein (AIRE), colonic and hepatic tumor overexpressed protein (CHTOG) and the gliomatumor suppressor candidate region gene 1 protein [23]. It has also been identified to be essential for mitogenic activity of PLCγ1 [24], as the SH3 domain, in combination with SH2 domains, induces mitogenesis in quiescent fibroblast, indicating its importance for cellular growth [25]. The interaction site for dynamin (a membrane-remodeling GTPase) is also located within PLCγ1-SH3 [26]. Pharmacological involvement of such interactions in various pathological conditions gives rise to the opportunity to identify therapeutic agents, specifically targeting the PLCγ1-SH3 and thus preventing the interaction between PLCγ1 and its cellular targets.

**Figure 1:**
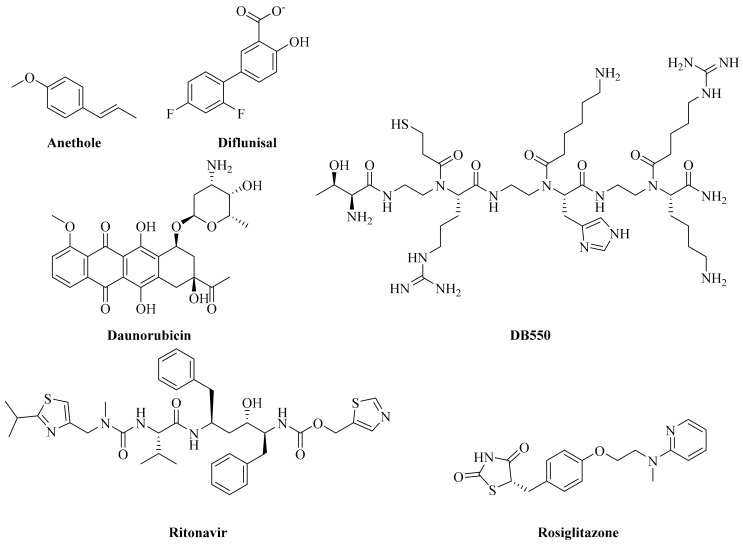
Structural formula of the reported inhibitors of phospholipase C gamma 1–cluster of differentiation 95 (PLCγ1–CD95) interactions [16].

The lymphocyte cytosolic protein 2, also known as SLP76 is a T-cell adaptor protein, which has been structurally characterized to interact with the PLCγ1-SH3 (PDB ID: 1YWO, Figure S1) [18], and thus offers the representative interactions between the PLCγ1-SH3 and its substrates. The PLCγ1-SH3 binds to the XPXXPXR motif of SLP76, which is more specific than a usual PXXP motif. Using this information, Poissonnier et al. have reported the design of an original peptidomimetic inhibitor, by employing molecular modeling studies [16]. In their work, protein-fragment complementation assay (PCA) and in vitro screening of 1280 molecules (Prestwick library) has been performed, identifying the inhibitors of PLCγ1–CD95 interactions which include ritonavir (HIV protease inhibitor), anethole (flavoring agent), daunorubicin (topoisomerase inhibitor), diflunisal (nonsteroidal anti-inflammatory drug), and rosiglitazone (antidiabetic agent which interacts with peroxisome proliferator-activated receptor) [16] (Figure 1). Additionally, a peptidomimetic (named DB550) (Figure 1) was designed on the basis of structural features extracted from the calcium inducing domain (CID) of CD95. These inhibitors were demonstrated to specifically inhibit the interactions of PLCγ1 and CD95. Administration of both ritonavir and DB550 showed therapeutic effects in lupus-prone mice [16]. Overall these findings indicate that targeting the PLCγ1-SH3 is of prime importance for the management of various pathological conditions involving a plethora of immunological conditions and cancers. Availability of three dimensional (3D) complex between various drug targets and their modulators become crucial for facilitating the rational drug design. Although, the experimental techniques, such as X-ray crystallography and NMR, are powerful tools in determining these structures, these is time-consuming and expensive, and not feasible for several proteins. The molecular modeling techniques such as homology modeling, molecular docking, and molecular dynamics simulations offer an appropriate solution for the prediction of intermolecular recognition interactions [9,10,11,27,28,29]. Several examples of successful application of molecular modeling techniques for the identification of potential therapeutic agents are available in literature [29,30,31,32,33,34,35,36]. In view of this, the computational methods were utilized in the current study. To the best of our knowledge, combining molecular docking and molecular dynamics (MD) simulations have never been performed on the PLCγ1-SH3.

In the present study, the PLCγ1-SLP76 complex (Figure S1) is therefore exploited for identifying novel inhibitors targeting PLCγ1 through a structure-based pharmacophore map to identify key structural features involved at the PLCγ1-SLP76 interface. As a first step, the molecular docking and molecular dynamics simulations of reference compounds, shown in Figure 1, is performed to characterize key residues as well as their binding affinity, so as to obtain reference values. Thereafter, a virtual library of compounds (constituted of 227,228 molecules) was subjected to a systematic virtual screening protocol, from which the top sixteen molecules were considered for an extended work using MD simulations to ensure their stable binding with PLCγ1. After a careful analysis of the MD results, it was found that out of the sixteen molecules, seven were highly promising candidates for inhibiting the interaction between the PLCγ1-SH3 and its target proteins. To the best of our knowledge, this is the first attempt to employ SLP76-based features for drug design against PLCγ1 as well as to screen such a large library of 227,228 compounds.

## 2. Results and Discussion

### 2.1. Molecular Recognition of SLP76 and Known Inhibitors by PLCγ1

**Figure 2:**
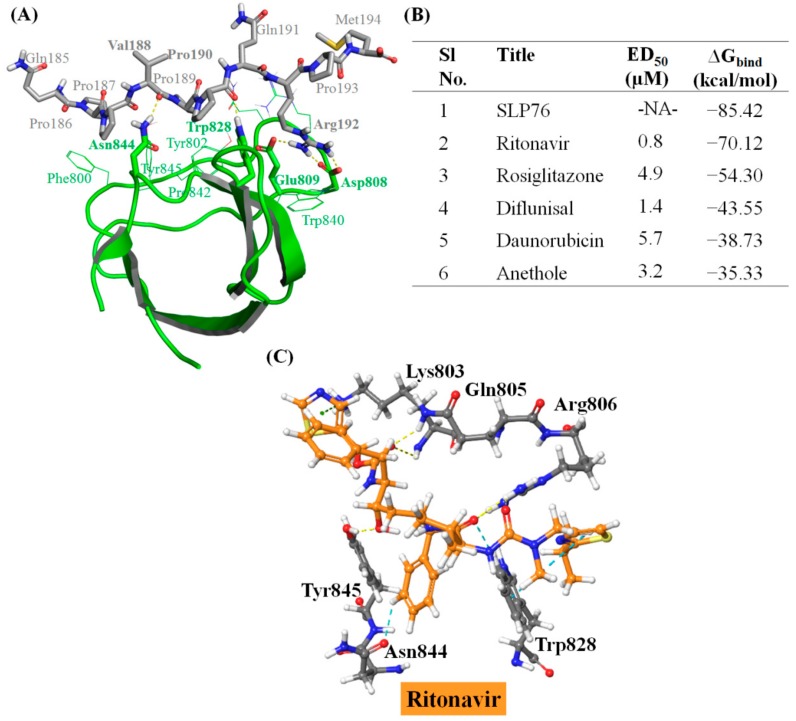
Interactions of SLP76 and reported inhibitors [16] with PLCγ1. (**A**) Main interactions between SLP76 and PLCγ1 in the X-ray crystal structure [18]. Residues, involved in H-bonds are shown in bold and stick. (**B**) Experimental ED_50_ values [16] and calculated MM/GBSA binding energy (∆G_bind_) for SLP76 (in the crystal structure) and reported inhibitors (after IFD) with PLCγ1. (**C**) Key interactions of ritonavir with PLCγ1. Legend for interactions: H-bonds in yellow; π···cation interactions in green; π···π stacking interactions in blue; aromatic H-bonds in cyan; salt bridges in magenta. -NA-: Not Applicable.

The PLCγ1-SLP76 crystal structure (PDB ID: 1YWO) [18] offers the opportunity to employ a structure-based drug design strategy for the identification of novel PLCγ1 inhibitors. Deng et al. employed isothermal titration calorimetry to identify a proline rich motif (^186^PPVPPQRP^193^) in SLP76 [18]. In the crystal structure, SLP76 forms four H-bonds with PLCγ1 via Asp808, Glu809, Trp828 and Asn844 (Figure 2A) and hydrophobic interactions at the protein–peptide interface through the proline enriched motif (XPXXPXR). Globally, the binding of SLP76 with PLCγ1 is governed by both, structural and electrostatic complementarity (Figure 2A and Figure S2A,B) [18]. Indeed, PLCγ1 possesses an arginine binding site which is characterized by a highly electronegative surface potential due to the presence of acidic Asp808 and Glu809 (Figure S2A). The latter are complementary to the highly electropositive Arg192 of SLP76 (Figure S2B) forming a salt bridge interaction. The ∆G_bind_ value for co-crystallized conformation of SLP76 with PLCγ1 was estimated through MM/GBSA calculation to a high magnitude value, i.e., −85.42 kcal/mol (Figure 2B).

Molecular docking of the reported PLCγ1 inhibitors (Figure 1) [16] was performed firstly to compare their binding values with SPL76 and secondly to establish selection criteria for the following step, that is the virtual screening. Structural superimposition of the predicted docked poses of these inhibitors in PLCγ1-SH3 reveals that all inhibitors overlap with the SLP76 peptide (Figure S3), especially at the C-terminal of SLP76. We do note, however, that ritonavir and rosiglitazone are slightly less aligned onto the SLP76 *N*-terminal side. ∆G_bind_ values were computed for each reported inhibitor so as to evaluate its correlation with their reported effective dose (ED_50_) [16] (Figure 2B). A direct correlation is rather difficult to establish between the ED_50_ and calculated ∆G_bind_ values, but it is clear that SLP76 and ritonavir have the highest binding affinity following the ED_50_ trend. Among the reported inhibitors, ritonavir is effectively the most potent inhibitor (ED_50_ of 0.8 µM and ∆G_bind_ of −70.12 kcal/mol) of the PLCγ1 and CD95 interaction. Table S1 enlists all the relevant non-covalent interactions between the reported inhibitors [16] and PLCγ1 the molecular docking (IFD) (see supporting information for details). 

### 2.2. Pharmacophore Mapping and Molecular Docking Based Identification of Promising Hits

**Figure 3:**
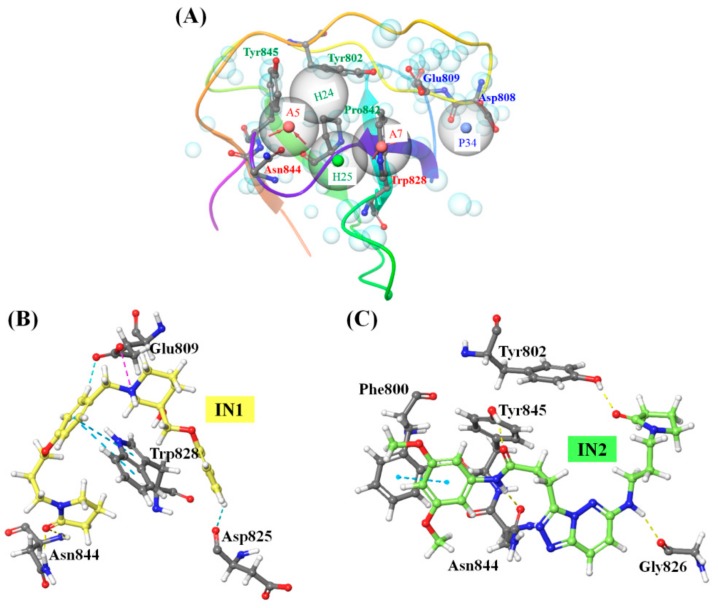
Results from the virtual screening performed for the identification of promising PLCγ1 inhibitors (**A**) Generated pharmacophore hypothesis from PDB ID: 1YWO [18]. Selected five pharmacophore features are shown as large grey spheres, where A: H-bond acceptor. The red arrows indicate the direction of H-bond formation (“A” being the H-bond acceptor); H: hydrophobic group; P: Positive functional group, which is treated equivalent to H-bond donor. Cyan spheres represent excluded receptor volume shell. (**B**) and (**C**) Non-covalent interactions of IN1 and IN2, respectively, with PLCγ1 (see Figure 2C for color legend).

Reported interactions between SLP76 and PLCγ1 (Figure 2A) can be considered as the important pharmacophoric features of PLCγ1 interacting agents, as also confirmed by the inhibitor binding (Table S1, Figure 2C and Figure S4). A structure-based pharmacophore map was created accordingly followed by a virtual screening helping to firstly identify compounds possessing similar SPL76 specific binding features. Out of a total of thirty-four structural, hydrophobic and electrostatic features present in SLP76 peptide, five of them were kept (Figure 3A) based on the interactions reported in literature [18], and observed in the available crystal structure (Figure 2A). The pharmacophore is thus built in order to selected compounds which contain two H-bond acceptors mimicking Val188 and Pro190, two hydrophobic groups aligning on Pro189 and Pro190 and one positive feature as Arg192 in SPL76 which was also treated as H-bond donor features. From the initial virtual library of compounds (227,228 molecules) used for the virtual screening, 2734 molecules simultaneously exhibit the five selected pharmacophore features (alignment of top 15 molecules with the generated pharmacophore is shown in Figure S5). Analysis of the topological diameter (range 10 to 25), molecular weight (>350 D) and molecular volume (>1000 Å^3^) of the 2734 molecules showed that they exhibit a large size (Figure S6A–C), which helps in occupying the ligand binding site as for the SPL76 peptide in the PLCγ1-SH3 and, possibly, could enhance the specificity of the molecule towards PLCγ1. The octanol/water partition coefficient for most of the selected molecules was in the range of 2 to 5 (Figure S6D) indicating their possible ability to permeate through membranes. Thereafter, a systematic molecular docking protocol (Figure S7) was employed to realize the interaction of the 2734 molecules with the PLCγ1-SH3. 

To narrow down the number of compounds a HTVS has been realized filtering compounds specifically interacting with the PLCγ1 arginine binding site (Asp808 and Glu809) and with the XPXXPXR proline enriched motif (Trp828 and Asn844) of PLCγ1. With the motive of blocking PLCγ1 activity, the presence of interactions within the arginine binding site and at least two H-bonds between screened molecules and PLCγ1 was considered as the selection criterion. Of these two H-bonds, one should be present with the arginine binding site (Asp808 or Glu809) and the other within the XPXXPXR motif recognition site (Trp828 or Asn844). The HTVS helped to filter this set of molecules to 705 compounds (with Glide gscore ≤ −3.5) which were subsequently subjected to molecular docking with higher precision. Evaluation of molecular docking results on the basis of glide gscore, reproducibility of docked conformation and most importantly, structural overlap with cocrystallized ligand SLP76, helped us to identify potential candidates for PLCγ1 inhibition. Final selection after each docking step was based on the calculated ∆G_bind_ value. SP mode and XP mode molecular docking (Figure S7) helped to bring the number of promising hits to 33 molecules characterized by ∆G_bind_ value below −40 kcal/mol, while IFD further narrowed down this number to 16 molecules (Table S2 and Figures S8 and S9). All molecules occupied the similar interaction site as one of the *N*-terminal domain of SLP76 (Figure S10). Molecular interactions between each ligand and PLCγ1 after the last molecular docking step (IFD) are presented in Table 1. 

The 3D molecular recognition interactions for top scoring hits, i.e., IN1 and IN2 are shown in Figure 3B,C, whereas interactions for the other 14 molecules (IN3-IN16) are shown in Figure S11. PLCγ1 residues which participated in H-bonds or salt bridge interactions with all the 16 selected ligands are Gln805, Asp808, Glu809, Trp828, Asn844, and Tyr845 (Table 1). Additional complex stabilization was observed pertaining to NH∙∙∙π /π∙∙∙π interactions with Arg806, Trp828, Trp840, and Tyr845. Hydrophobic interactions were mainly observed with Tyr802, Gly826, Gly827, Trp840, Phe841, Pro842, and Tyr845, for each ligand. As discussed earlier, a careful attention was paid throughout the molecular docking steps to keep two key intermolecular interactions, i.e., at arginine binding site and proline (from XPXXPXR motif) binding site. The ∆G_bind_ values calculated after IFD (Table S3) were comparable to that of ritonavir and SLP76 (Figure 2B), ranging from −78.07 to −56.34 kcal/mol, thus further supporting their candidature as PLCγ1 inhibitors. Interestingly, all selected compounds possessed a basic nature (predicted *pKa* value > 13), facilitating their interaction with the negatively charged arginine binding site of PLCγ1. As shown in Figure 3B,C and Figure S11, a positively charged nitrogen center in these molecules occupied the arginine binding site by interacting with Asp808 or Glu809. These generated complexes were thus taken further for the MD simulations.

### 2.3. MD Simulations

In order to evaluate the stability of the identified interactions under dynamical conditions and ensure strong binding of ligands with the target, MD simulation is a method of key choice. The generated 16 complexes were submitted to MD simulations for 50 ns to study the system relaxation. Additionally, PLCγ1-SLP76 and PLCγ1-ritonavir complexes were also subjected to MD simulations, as they are considered as reference systems. To ensure reproducibility of the results, each system was simulated in three replicates. Combined cluster analysis (Figure S12) revealed that three replicates behave similarly (keeping 70% as cut-off) for the complexes formed by SLP76, IN1, IN6, IN11, and IN15 with PLCγ1. Indeed, the majority of the three simulation coordinates belongs to one unique cluster. For the systems containing ritonavir, IN2, IN3, IN4, IN5, IN7, IN9, IN12, and IN13, the cluster population was spanned over two clusters, while for PLCγ1 bound to IN8, IN10, IN14, and IN16, at least one of the simulations indicated a wider distribution of the cluster population over the period of simulation run. The RMSD analysis between the clusters in the various systems showed that the inter-cluster distance was <2 Å (Table S4) and the average distance from the centroid for various clusters was <1.5 Å for all the systems. Thus, it can be concluded that the triplicate simulations successfully produced comparable results.

In order to evaluate the stabilized binding of each ligand to PLCγ1 in the generated complexes over the period of simulation, the distribution of each cluster population with time was analyzed (Figure S13). For SLP76, ritonavir, IN1 to IN5, IN8, IN11, and IN13 to IN16, >70% of the frames remained in a single cluster over the last 25 ns of simulation in the three replicate runs. For IN6, IN7, IN10, and IN12, at least two replicates showed an equilibrated trajectory over the entire simulation run. The whole protein RMSD analysis showed that PLCγ1 structure was stabilized during the simulation and showed minimum difference (RMSD < 2.0 Å) in the various complexes as compared to their initial coordinates (Figure S14). 

After the global evaluation of the simulation trajectories, we decided to analyze the local behaviors of the molecules at the binding sites. The structural overlap of the ligand position after molecular docking, after the system equilibration and after the production run (for one representative replicate) is shown in Figure S15. For SLP76, the structure overlap was performed between the cocrystallized conformation, equilibrated conformation and the structure after MD simulations. The three structures indicate a clear overlap between each SLP76 conformation (Figure S15A) which indicates the ability of the adopted protocol to maintain the cocrystallized conformation. Compared to the complex generated after molecular docking, the position of the ligand did not change much after system equilibration for majority of the 16 molecules, except for ritonavir and IN11. After the MD simulations, IN1, IN4-IN6, IN8, IN10, and IN13-IN16, were maintained close to the docked pose. The final structure after the MD simulation revealed a significant movement from the docked position for ritonavir, IN3, IN7, IN11, and IN12, while for others no major change in their relative position was observed (Figure S15). For compound IN3, this displacement was mainly observed in the position of pyrazolo (3, 4-*d*) pyrimidinyl ring from the arginine binding site to Proline motif binding site. For IN7, IN11 and IN12 an obvious unbinding of the compound from PLCγ1 was observed during each replica of the molecular dynamics simulations. For a detailed investigation of binding behavior of the identified hits, the RMSD and distance from crucial residues were evaluated (Figures S16 and S17).

### 2.4. Stable Binding of Identified Molecules to PLCγ1

In order to select the molecules which showed reproducible and stable binding to PLCγ1, RMSD along the simulations and time-dependent distance between the center of mass of the bound ligand and Asn844 of PLCγ1 (COM_dist) were plotted to rapidly identify the unbinding of some ligands (Figures S16 and S17). The cocrystallized peptide SLP76 shows a very stable complexation with PLCγ1 throughout all the simulation runs, as indicated by the stable RMSD value for the entire complex, by the COM_dist and by the ligand RMSD (Figure S16A). Structural overlap of the final coordinates for the three replicates of PLCγ1-SLP76 complex indicates a similar orientation of SLP76 in the binding site, except for its terminal amino acids (Gln185 and Met194). The SLP76 position after MD simulation is highly similar to the one from cocrystallized conformation (RMSD values ranging from 2 to 4 Å). Additionally, the calculated per-nanosecond ∆G_bind_ value for the PLCγ1-SLP76 system indicates a stabilized affinity along the simulation run (Figure S18) with an average ∆G_bind_ value (over last 5 ns) of −50.14 ± 3.96 kcal/mol (Table S5). It can be observed that compared to the ∆G_bind_ value from MM/GBSA calculations (Figure 2B), the value after MD simulations (Table S5) is numerically increased significantly from −85 kcal/mol to −50.14 ± 3.96 kcal/mol, respectively, signifying lowered affinity. From the component analysis of ∆G_bind_ (vdW, electrostatic, etc.), the complexation is dominated by the electrostatic component (−119.92 kcal/mol, Table S5) which is attributed to the strong interaction at the arginine binding site.

The PLCγ1-ritonavir complex generated from the IFD (Figure 2C) was also submitted to MD simulations, which resulted into the stabilized PLCγ1-ritonavir complex (RMSD and COM_dist in Figure S16B), and here again, the calculated ∆G_bind_ value for three replicates (Figure S18) were numerically increased (−28.42 ± 3.31 kcal/mol in Table S5) compared to the one after IFD (−70.12 kcal/mol in Figure 2B), which is much higher (lower affinity) than the ∆G_bind_ of SLP76 (difference of 21.72 kcal/mol). Such a variation can be attributed to the difference in molecular dimension and surface electrostatics between both partners. As we know, SLP76 is an intracellularly present binding partner for PLCγ1, while ritonavir is required to cross the membrane barrier for interacting with its macromolecular drug targets and its smaller molecular size favorably contributes to penetrate the cell membrane. Therefore, normalized ∆G_bind_ value (based on molecular weight or molecular volume) (Table S6) were used to obtain a ∆G_bind_ values accounting for such bias. ∆G_bind_ per unit weight (ΔG_bind-MW_) and per unit volume (ΔG_bind-MV_) for SLP76 are calculated to be −0.044 kcal/mol and −0.015 kcal/mol, respectively, while for ritonavir ΔG_bind-MW_ and ΔG_bind-MV_ are −0.040 kcal/mol and −0.013 kcal/mol, respectively. Such normalized ∆G_bind_ values show, as expected, that the binding of ritonavir is highly comparable to SLP76. As ritonavir has been already reported to inhibit the interaction of PLCγ1 with the CD95 death domain by binding at PLCγ1-SH3 [16], we consider the ∆G_bind_ value of ritonavir as a cutoff for the selection of potent inhibitors.

**Figure 4:**
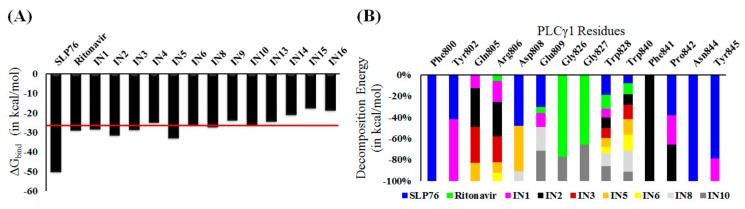
Interaction analysis for the molecular dynamics of the sixteen complexes. (**A**) Average ∆G_bind_ value calculated over the last 5 ns. Red line indicates the cutoff used for final selection of compounds and (**B**) Per-residue decomposition energy analysis for selected potential PLGγ1-inhibtior complexes during molecular dynamics (MD) simulation.

Based on the RMSD and COM_dist values (Figures S16 and S17), molecules showing stable binding to PLCγ1 can be rapidly identified. Three ligands out of the selected 16 compounds, IN7, IN11 and IN12, were released from the binding site of PLCγ1 in at least one of the replicate simulation runs (Figures S16I and S17D,E). Other ligands remained bound at the PLCγ1-SH3. Analysis of ∆G_bind_ over the last 10 ns simulation trajectory (Figure S18) helped to identify molecules with similar binding behavior with PLCγ1 as that of ritonavir. Molecules for which ∆G_bind_ was numerically lower than −25 kcal/mol in all replica simulations (Figure S18) are IN1, IN2, IN3, IN5, IN6, IN8, and IN10 and can be considered equivalent in terms of ∆G_bind_ to ritonavir (Figure 4A and Table S5). Interestingly, ∆G_bind-MW_ and ∆G_bind-MV_ for all the selected molecules (except for IN7, IN11, and IN12, which were released from the binding site) were lower than SLP76 and ritonavir (Table S5) indicating even a stronger binding towards PLCγ1. The per-residue atomic fluctuation for each system also indicates that the molecules which showed stable binding also present lower fluctuation in PLCγ1 (Figure S19). Contrarily, IN7, IN11, and IN12, which are released from the binding site, induced a higher degree of structural fluctuation within the protein structure. Hereafter, IN7, IN11, and IN12 were not considered for the rest of the analysis.

### 2.5. Molecular Recognition of the Selected Molecules to PLCγ1 Considering MD Simulations

Molecular recognition interactions play a crucial role in ensuring stability of the complex and binding affinity of the molecules to their target. An analysis of per-residue total decomposition energy allowed the identification of key amino acids involved in favorable interactions with ligands. Residues with a significant contribution to the binding energy (cutoff −0.5 kcal/mol) involving the ligands and PLCγ1 are presented as stacked bar plot in Figure 4B. The critical residues for SLP76 binding to PLCγ1-SH3 were Phe800, Tyr802, Asp808, Glu809, Trp828, Trp840, Pro842, Asn844, and Tyr845. Interestingly, these results were in good correlation with the reported important residues (Asp808, Trp840, and Tyr845) (detected from NMR data) [16] for interaction of SLP76 with PLCγ1-SH3. Residues which were identified to be involved in interaction with inhibitors are Gln805, Arg806, Asp808, Glu809, Trp828, and Trp840. Such conclusion was based on their involvement in the complex formation for multiple systems (indicated by presence of multiple colors in the stacked bars). Interestingly, these residues also exhibited lower atomic fluctuation in the presence of bound ligands (Figure S20), and were also reported to be crucial for SLP76 binding to PLCγ1 [18]. Thus, their involvement in interaction with identified compounds, increases the confidence in PLCγ1 inhibiting ability of the selected molecules.

**Figure 5:**
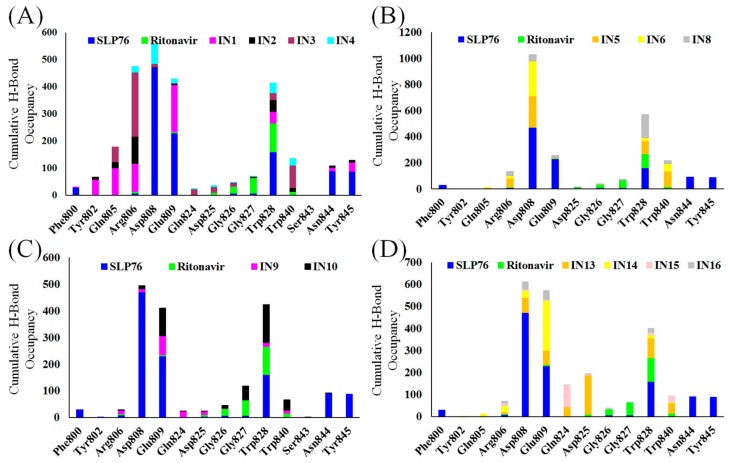
H-bond occupancy analysis for the PLCγ1 residues in various systems over the last 10 ns in various complexes after MD simulations.

Residues which were involved in H-bond interactions with the selected molecules were identified by analyzing the last 10 ns MD simulation trajectory for the various complexes. Average number of H-bonds (Figure S21) were found to be higher than 3 for IN1, IN3, IN5, IN6, IN10, and IN13 (Table S7). Cumulative H-bond occupancy analysis (Figure 5) helped to identify the residues involved in H-bond interactions. Stacked bars with a high degree of color variation (indicating the presence of H-bond in several PLCγ1-inhibitor complexes) represent the residues important for stabilization of PLCγ1-inhibitor complex, which include Arg806, Asp808, Glu809, and Trp828. These residues were also found to interact with SLP76 during MD simulations via H-bonds. Residues Gln805, Asp825, and Gly827 were involved in H-bond interactions with the inhibitors, but not with SLP76. Their importance in inhibitor binding can be evaluated in vitro; however, this is not covered in the scope of current study.

**Figure 6:**
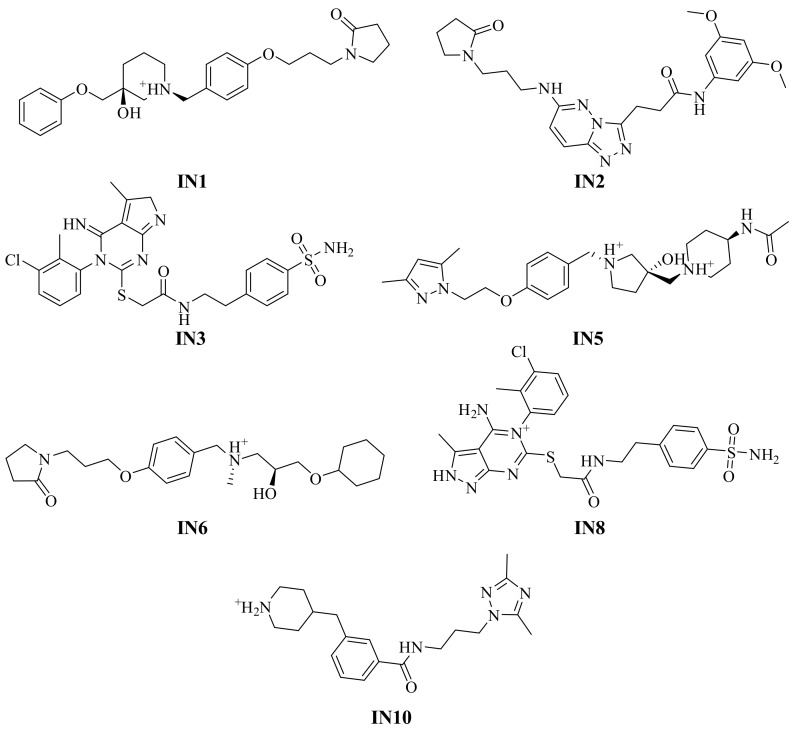
Selected potential inhibitors PLCγ1 after the three replicate MD simulations.

Based on the extensive MD simulation analysis, performed herein, IN1, IN2, IN3, IN5, IN6, IN8, and IN10 (Figure 6) were proposed as the most potent candidates for PLCγ1 inhibition (Table S2). At the moment, none of these molecules have been evaluated for any kind of biological activity according to the ChEMBL database [37], making those highly interesting compounds for further developments. In vitro evaluation of their binding to PLCγ1 and subsequent, interference in the interaction of PLCγ1 with its cellular targets would be of great therapeutic relevance.

## 3. Materials and Methods

### 3.1. Pharmacophore Modeling

Starting from the available crystallographic structure of PLCγ1-SLP76 complex (PDB ID: 1YWO) [18], a pharmacophore model was defined based on all potential pharmacophore features of the ligand complementary to the substrate-binding site using the PHASE module [38,39] of the Schrödinger software package, version 2018-2 [40]. Structural features of SLP76 which facilitate its binding to the PLCγ1-SH3 were identified and five of them were selected from a collection of fourteen features. An excluded volume shell based on van der Waals radii was also taken into consideration to mimic the receptor binding site while generating the pharmacophore model. Eventually, from the 227,228 compounds arising from various libraries (Table S8), 2734 molecules exhibit simultaneously the five features defined by the pharmacophore model. These were subjected to molecular docking.

### 3.2. Molecular Docking-Based Virtual Screening

The crystal structure of the complex between the PLCγ1-SH3 (from *Rattus norvegicus*) and SLP76 (from *Homo sapiens*) (PDB ID: 1YWO) [18] was considered for molecular docking. High sequence identity (95%) and sequence similarity (98%) between the PLCγ1-SH3 from *Rattus norvegicus* (UniProt ID: P10686) and from *Homo sapiens* (UniProt ID: P19174) (Figure S22A) permits the use of such structure for the molecular modeling studies. Indeed, the three non-identical residues of PLCγ1 (Ile813, Glu825 and Ile846 in *Homo sapiens* and Thr813, Asp825 and Val846 in *R. norvegicus*) are not involved in the interaction with SLP76 (Figure S22B,C). Using the Protein Preparation Wizard module [41] of Schrödinger software package, version 2018-2 [42], pre-processing of the macromolecular structure was performed, i.e., addition of missing hydrogens, removal of water molecules beyond 5 Å and assignment of the right bond order. The *protassign* utility of the Protein Preparation Wizard module was employed for optimization of ionization state using PROPKA, for predicting p*Ka* values in proteins (pH 7.0 ± 2.0) and orientations of side chain functional groups (e.g., hydroxy group in Ser, Thr and Tyr; amino group in Asn and Gln). A restrained minimization of the complex was then performed (cutoff root mean square deviation (RMSD) 0.3 Å) with the help of *impref* utility, so as to avoid any steric clashes.

The 2734 molecules obtained after the pharmacophore filtering were prepared using the LigPrep module of Schrödinger software package, version 2018-2 [41,43]. For the high-throughput virtual screening (HTVS) step, the ionization states of these molecules were not considered, whereas for subsequent steps, these molecules were subjected to preparation in LigPrep, generating their ionization states (using Epik ionizer [44,45,46], pH 7.0 ± 2.0). For a comparative analysis, reported PLCγ1 inhibitors [16], i.e., anethole, daunorubicin, diflunisal, ritonavir and rosiglitazone (Figure 1) were also considered and submitted to molecular docking at the SLP76 binding site in PLCγ1-SH3.

The interaction grid for molecular docking was generated with the Receptor Grid Generation module of Schrödinger software package at the centroid of bound ligand in the prepared PLCγ1-SLP76 complex (grid center: 19.29, 2.63, 25.99). The size of the interaction grid was extended up to 10 Å as inner box and additional 20 Å as outer box. Molecular docking was performed using the Glide module of Schrödinger software package [47,48,49,50] in four steps (Figure S7), i.e., (i) high-throughput virtual screening (HTVS), (ii) Standard Precision (SP) mode docking, (iii) eXtra Precision (XP) mode docking and (iv) Induced Fit Docking (IFD) [51,52,53]. For HTVS, only one pose was considered, whereas for subsequent steps, 20 poses were generated for each molecule (with all parameters at their default values and by employing the OPLS_2005 force field) [54]. After each step, results were subjected to a pose filtering for the presence of crucial hydrogen bond (H-bond) interactions with PLCγ1 (via Asp808/Glu809 and Trp828/Asn844), evaluation for structural overlap with cocrystallized ligand, reproducibility of the docked conformation and glide docking score. Molecular Mechanics-Generalized Born Surface Area (MM/GBSA) based binding free energy (∆G_bind_) were computed for the complexation of selected molecules with PLCγ1, using Prime module [55]. Molecules with a ∆G_bind_ value lower than −40 kcal/mol were taken for next steps. For the hit selection after IFD, the ∆G_bind_ cutoff was kept to −55 kcal/mol. The sixteen molecules, named hereafter as INX (where X = 1 to 16) were further considered for the MD simulations. Previously reported PLCγ1 inhibitors [16] (Figure 1) were also submitted to IFD and, subsequent MM/GBSA ∆G_bind_ calculations.

### 3.3. Molecular Dynamics Simulations

In order to evaluate the stability of sixteen complexes generated from molecular docking, PLCγ1-ritonavir complex and PLCγ1-SLP76 complex, MD simulations were carried out using the AMBER18 package [56]. The General Amber Force Field (GAFF) [57] and Amber ff99SB force field [58] were employed for ligands and protein preparation, respectively. The AM1-bcc method (semi-empirical with bond charge correction) [59] of the antechamber module from Amber tools 18 [56] was utilized for deriving charges on the ligands. TIP3P water model [60] was used for solvation (cubic box; 15 Å × 15 Å × 15 Å). Each system was neutralized by adding counter ions and an ionic concentration of 0.15 M was maintained by adding additional Na^+^ and Cl^−^ ions. All systems were subjected to minimization and gradual heating (from 0 to 300 K, under NVT ensemble). Thereafter, density equilibration (under NPT ensemble) and equilibration (1 ns under NPT ensemble) were performed sequentially at 310 K and 1 atm pressure (pressure relaxation time of 2.0 ps and time step of 2 fs). Finally, three replica of the production run for 50 ns were performed under NPT ensemble for each system using a cutoff distance of 12 Å for non-bonded interactions. Long-range electrostatic interactions were treated with the Particle-Mesh Ewald (PME) method [61]. Bulk effect was simulated by enabling periodic boundary conditions. All covalent bonds containing hydrogen atoms were constrained using the SHAKE algorithm [62]. Ptraj module [63] of Amber tools [56] and Visual Molecular Dynamics software (VMD) [64] were used for trajectory analysis. Combined clustering analysis was performed, for the three replicate MD simulations, Ptraj module [63] to evaluate the reproducibility of the results and ligand binding during the simulation. A hierarchical agglomerative (bottom-up) approach was employed as clustering algorithm (number of clusters: 5) and the best-fit coordinate RMSD between all the heavy atoms was considered as the parameter for clustering. ∆G_bind_ values were also calculated using MM/GBSA method [65] over the last 10 ns of MD simulations trajectory.

## 4. Conclusions

Involvement of PLCγ1 in a number of cellular processes makes it an important drug target for a number of pathological and disease conditions, including immunological disorders and cancers. The PLCγ1-SH3 is known to be involved in interaction with several proteins, regulating a number of cellular processes. It has been proposed as an important target domain for the design of anti-PLCγ1 agents. The occupied binding site of PLCγ1-SH3 prevents the interaction of PLCγ1 with the target adaptor proteins, thus leading to the modification of cellular responses including cell proliferation, differentiation of cell death. Therefore, identification of compounds which can efficiently and stably bind to PLCγ1-SH3 was undertaken through computer aided drug design (CADD) study.

A systematic virtual screening was performed by employing a pharmacophore mapping based on the SLP76 peptide, molecular docking and molecular dynamics (MD) simulations. In this process, a large collection of 227,228 compounds was evaluated against the pharmacophore filtering which helped to identify 2734 compounds with potential features to bind at the PLCγ1-SH3. These molecules were then submitted to molecular docking in an increasing degree of precision, shortlisting sixteen compounds. Under static conditions, they exhibited a significant degree of binding affinity and important molecular recognitions with the PLCγ1. To evaluate the binding of the identified hits to PLCγ1 under dynamical conditions, MD simulations in triplicate were undertaken for each of the 16 complexes. System stability and binding energy analyses helped to identify compounds IN1, IN2, IN3, IN5, IN6, IN8, and IN10 (Figure 6) as promising candidates for inhibiting the interaction of PLCγ1 with its target proteins as they exhibit a stable binding at PLCγ1-SH3. Additionally, identified important molecular recognitions can help to streamline drug discovery against PLCγ1. Residues which participated in the stable binding of inhibitors to the protein are Gln805, Arg806, Asp808, Glu809, Asp825, Gly827, and Trp828. These results are in agreement with the reported experimental data [16]. To the best of our knowledge, this work is the first report of a systematic application of CADD for identification of inhibitors against PLCγ1. These molecules can be taken up further for in vitro evaluation of their PLCγ1 inhibiting effect.

## Figures and Tables

**Table 1:** Molecular recognition interactions between IN1-IN16 molecules and PLCγ1 after induced fit molecular docking. Residues in bold are also involved in similar interactions with SLP76.

Title	H-Bond	NH···π/π···π Stacking Interactions	Hydrophobic Interactions	Other Residues within 5Å
IN1	**Glu809**, **Asn844**	**Trp828**	Tyr802, Gly826, Gly827, **Trp840**, Phe841, **Pro842**, **Tyr845**	**Gln805**, **Arg806**, Asp808, Gln824, Asp825, Ser843
IN2	Phe800, Tyr802, Gly826, **Asn844**	**Tyr845**	Leu799, Gly827, Trp828, **Trp840**, Phe841, **Pro842**	**Gln805**, **Arg806**, Asp808, Glu809
IN3	Arg806, **Asp808**, Gly826, **Asn844**	**Trp828**, Trp840	Tyr802, Gly825, Phe841, **Pro842**, **Tyr845**	**Gln805**, Glu809, Ser843
IN4	Gln805, Arg806, **Asp808**, **Glu809**, Tyr845		Tyr802, Trp828, **Trp840**, **Pro842**, **Tyr845**,	Lys803, Glu807, Gln824, Asp825, Asn844
IN5	**Asp808**, **Glu809 Trp828**	Trp840	Tyr802, Gly827, Gly826, Trp829, Phe841, **Pro842**, **Tyr845**	**Gln805**, **Arg806**, Gln824, Asp825, Arg830, Ser843, Asn844
IN6	Gln805, Arg806, **Asp808**, Trp840		Tyr802, Trp828, Gly826, Gly827	Gln824, Asp825, Arg830
IN7	**Glu809**, Gly826	**Trp828**, Trp840	**Pro842**, **Tyr845**, Tyr802, Gly827	**Gln805**, **Arg806**, Asp808, Asp825, Ser843, Asn844
IN8	Arg806, **Asp808**, **Asn844**	**Trp828**, Trp840	Tyr802, Gly826, Gly827, Phe841, **Pro842**, **Tyr845**	**Gln805**, Glu809, Gln824, Asp825, Ser843
IN9	Asp808, **Glu809**, **Trp828**, Trp840	Arg806, Trp840	Tyr802, **Pro842**, **Tyr845**	**Gln805**
IN10	**Asp808**, Asn844	Trp840, **Trp828**	**Tyr845**, Tyr802, Gly826, Gly827, **Pro842**	**Arg806**, Glu809, Gln824, Asp825, Ser843
IN11	Gln805, **Trp828**, **Asn844**,		Tyr802, Gly826, Gly827, **Trp840**, **Pro842**, **Tyr845**	Lys803, **Arg806**, Asp808, Glu809, Arg830, Ser843,
IN12	Gln805, Arg806, **Glu809**	**Trp828**	Tyr802, Gly826, **Trp840**, **Pro842**, **Tyr845**	Asp801, Lys803, Asp808, Gln824, Asp825, Asn844
IN13	**Asp808**, Gly826, **Trp828**	**Trp828**, Trp840	Tyr802, Gly827, **Pro842**, **Tyr845**,	**Arg806**, Glu809, Asp825, Ser843, Asn844
IN14	**Asp808**, **Glu809**, Gly826	**Trp828**	Tyr802, Gly827, **Trp840**	**Arg806**, Gln824, Asp825
IN15	**Asp808**	**Trp828**, Trp840	Tyr802, Gly826, Phe841, **Pro842**	**Gln805**, **Arg806**, Glu809, Asp825
IN16	**Asp808**, Gly826, **Asn844**	**Trp828**	Gly827, **Trp840**, **Pro842**, **Tyr845**	**Gln805**, **Arg806**, Glu809, Asp825, Ser843

## Supplementary Materials

Supplementary materials can be found at https://www.mdpi.com/1422-0067/20/19/4721/s1.

## Abbreviations

3D	Three Dimensional
∆G_bind_	Binding energy
AMBER	Assisted Model Building with Energy Refinement
CADD	Computer Aided Drug Design
CD95	Cluster of differentiation 95
CID	Calcium inducing domain
COM_dist	Center of Mass of the Bound Ligand and AsnA844 of PLCγ1
GAFF	General Amber Force Field
H-bond	Hydrogen-bond
HTVS:	High-throughput virtual screening
IFD	Induced fit docking
IP3	Inositol 1,4,5-triphosphate
MD	Molecular dynamics
MM/GBSA	Molecular Mechanics-Generalized Born Surface Area
PDB	Protein Data bank
PIP2	Phosphatidylinositol 4,5-bisphosphate
PLCγ1	Phospholipase C gamma 1
PLCγ1-SH3	SH3 domain of PLCγ1
PME	Particle-Mesh Ewald
RMSD	Root mean square deviation
SP	Standard Precision
TIP3P	Three-site Transferrable Intermolecular Potential
VMD	Visual Molecular Dynamics software
XP	eXtra Precision
