# Supplementary material for: Investigation of Phospholipase Cγ1 Interaction with SLP76 Using Molecular Modeling Methods for Identifying Novel Inhibitors
Source: Int J Mol Sci. 2019 Sep 23;20(19):4721. doi: 10.3390/ijms20194721 (PMC6801593; doi:10.3390/ijms20194721)
Supplement: Supplementary file 1 [file ijms-20-04721-s001.pdf]

Supporting Materials

Investigation of Phospholipase Cγ1 Interaction with SLP76 Using Molecular Modeling Methods for Identifying Novel Inhibitors

Neha Tripathi [1], Iyanar Vetrivel [1], Stéphane Téletchéa [2], Mickaël Jean [3], Patrick Legembre [3,4] and Adèle D. Laurent [1,*]

[1] CEISAM UMR CNRS 6230, UFR Sciences et Techniques, Université de Nantes, 44322 Nantes Cedex 3, France; neha.tripathi@univ-nantes.fr (N.T.); iyanar.vetrivel@univ-nantes.fr (I.V.)

[2] UFIP UMR CNRS 6286, UFR Sciences et Techniques, Université de Nantes, 44322 Nantes Cedex 3, France; stephane.teletchea@univ-nantes.fr

[3] CLCC Eugène Marquis, Equipe Ligue Contre Le Cancer, 35042 Rennes, France; mickael.jean@univ-rennes1.fr (M.J.); patrick.legembre@inserm.fr (P.L.)

[4] COSS INSERM UMR1242, Université Rennes 1, 35042 Rennes, France

* Correspondence: Adele.Laurent@univ-nantes.fr; Tel.: +33-(0)251-125-743

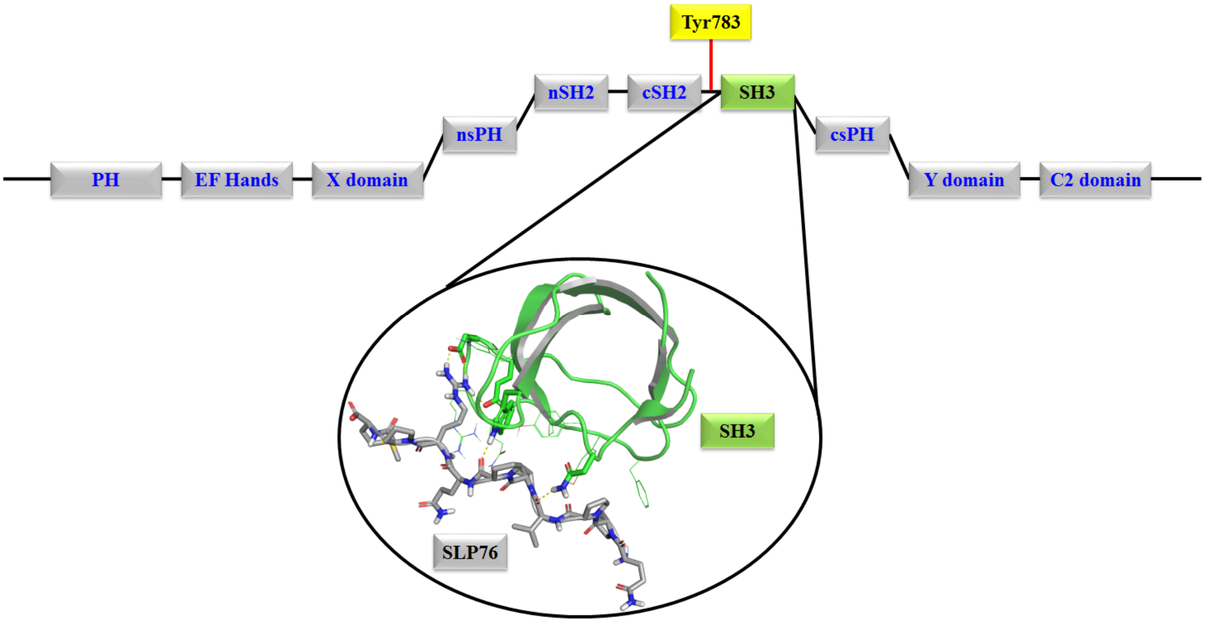

Figure S1. Structural topology of PLCγ1. PLCγ1 is composed of pleckstrin homology (PH) domain followed by four tandem EF hand domains, a split TIM barrel (which is an α/β protein fold) and a C2 domain [1,2]. PLCγ1 TIM barrel is the structural location for the catalytic site and calcium binding domain [3]. The TIM barrel is formed by X domain, followed by an N-terminal of split PH domain (nsPH), two SH2 (C-terminal SH2 and N-terminal SH2) domains, a SH3 domain, a C-terminal of split PH domain (csPH) and a Y domain [4]. The X and Y domains form the catalytic site of enzyme. XY linker is the differentiating feature between subfamilies. Complex containing the SH3 domain of PLCγ1 and SLP76 (PDB ID: 1YWO; resolution: 1.81 Å; R-Value Free: 0.221; R-Value Work: 0.171) [5] is shown in the inset. Site of phosphorylation, Tyr793, is shown in yellow box.

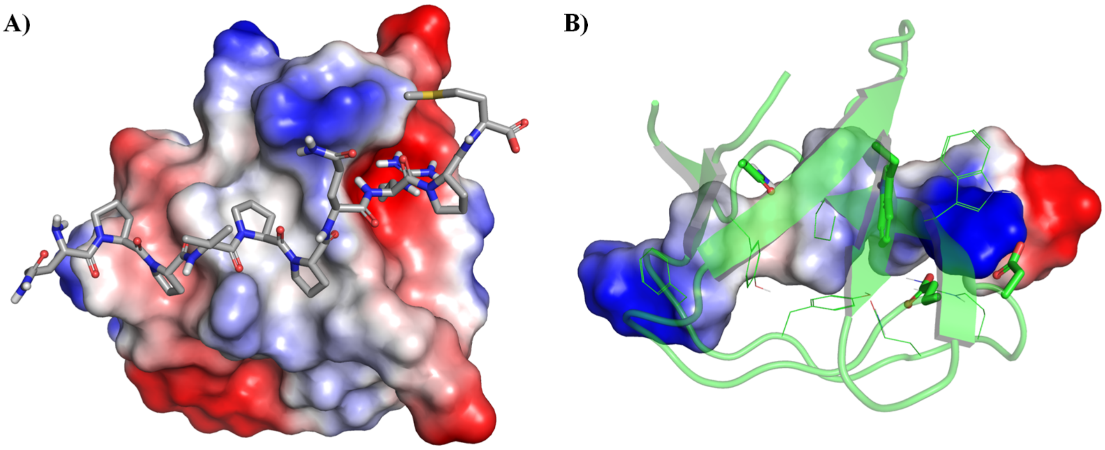

Figure S2. Structural details of PLCγ1-SLP76 complex (PDB ID: 1YWO) [5]. (A) Electrostatic surface potential of PLCγ1 (SLP76 is shown as sticks representation) and (B) SLP76 (PLCγ1 is shown as cartoon representation). Legend for surface coloring: blue, red and white represent the electropositive, electronegative and electroneutral surfaces, respectively.

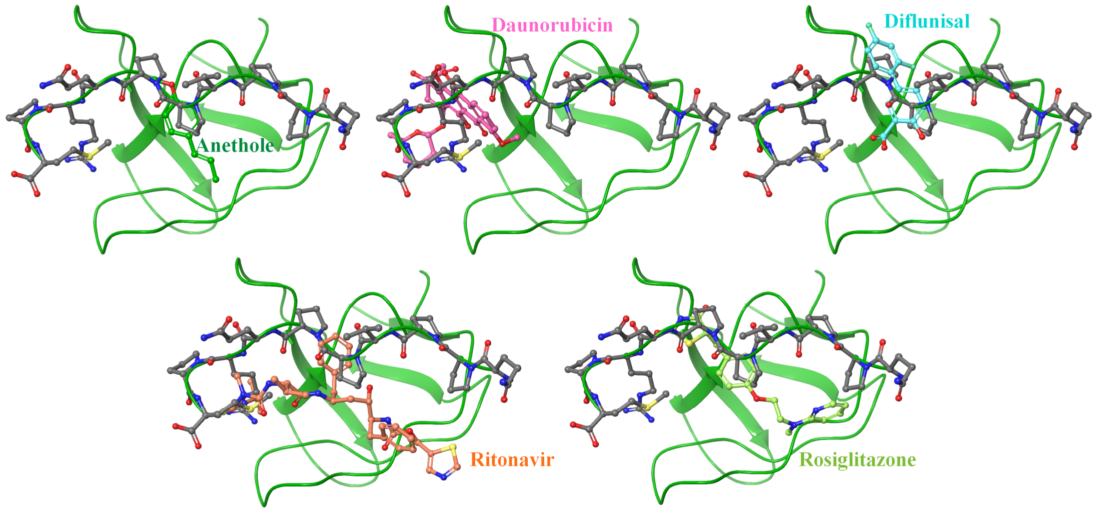

Figure S3. Structural overlap of reported PLGγ1 inhibitors [6] with SLP76 (grey) after induced fit molecular docking.

Important molecular recognition interactions between ritonavir and PLCγ1 originated to Gln805, Arg806, Tyr845 *via* H-bonds, to Lys803 *via* NH···π interactions and to Trp828 *via* π···π stacking interactions (Figure 2C in the main manuscript). Additionally, several hydrophobic interactions (Phe800, Tyr802, Gly826, Trp840 and Pro842) and polar interactions (Asp801, Asp808, Glu809 and Asn844) further stabilized the generated PLCγ1-ritonavir complex. The crucial H-bond interactions were also observed between other reported inhibitors and PLCγ1 at the C-terminal (Table S1 and Figure S4). PLCγ1-inhibitor complexes were all characterized by π···π stacking interactions with Trp828. After such a careful analysis, the key interactions playing a pivotal role in the recognition by PLCγ1 were thus further utilized to identify potential candidates for PLCγ1 inhibition.

Table S1. Non-covalent interactions of SLP76 (in the crystal structure) and the reported inhibitors [6] (after induced fit docking) with PLCγ1.

Title	H-bond/Salt-bridge interactions	NH···π/ CH···π/ π···π stacking interactions	Hydrophobic interactions	Polar interactions
SLP76	Asp808, Glu809, Trp828, Asn844		Phe800, Tyr802, Pro842, Tyr845	Gln805, Arg806
Anethole	Asn844	Trp828	Tyr802, Ala804, Pro842, Tyr845	Asp801, Lys803, Gln805, Arg806, Ser843
Daunorubicin	Arg806, Asp808, Glu809, Trp828		Tyr802, Gly826, Gly827, Trp829, Pro842	Gln805, Gln824, Asp825, Arg830
Diflunisal	Gln805, Arg806, Trp828		Tyr802, Gly826, Gly827, Pro842, Tyr845	Glu809, Ser843, Asn844
Rosiglitazone	Gln805, Gly826, Trp828	Tyr845	Phe800, Tyr802, Gly827, Pro842	Asp801, Lys803, Ser843, Asn844
Ritonavir	Gln805, Arg806, Tyr845	Lys803, Trp828	Phe800, Tyr802, Gly826, Trp840, Pro842	Asp801, Asp808, Glu809, Asn844

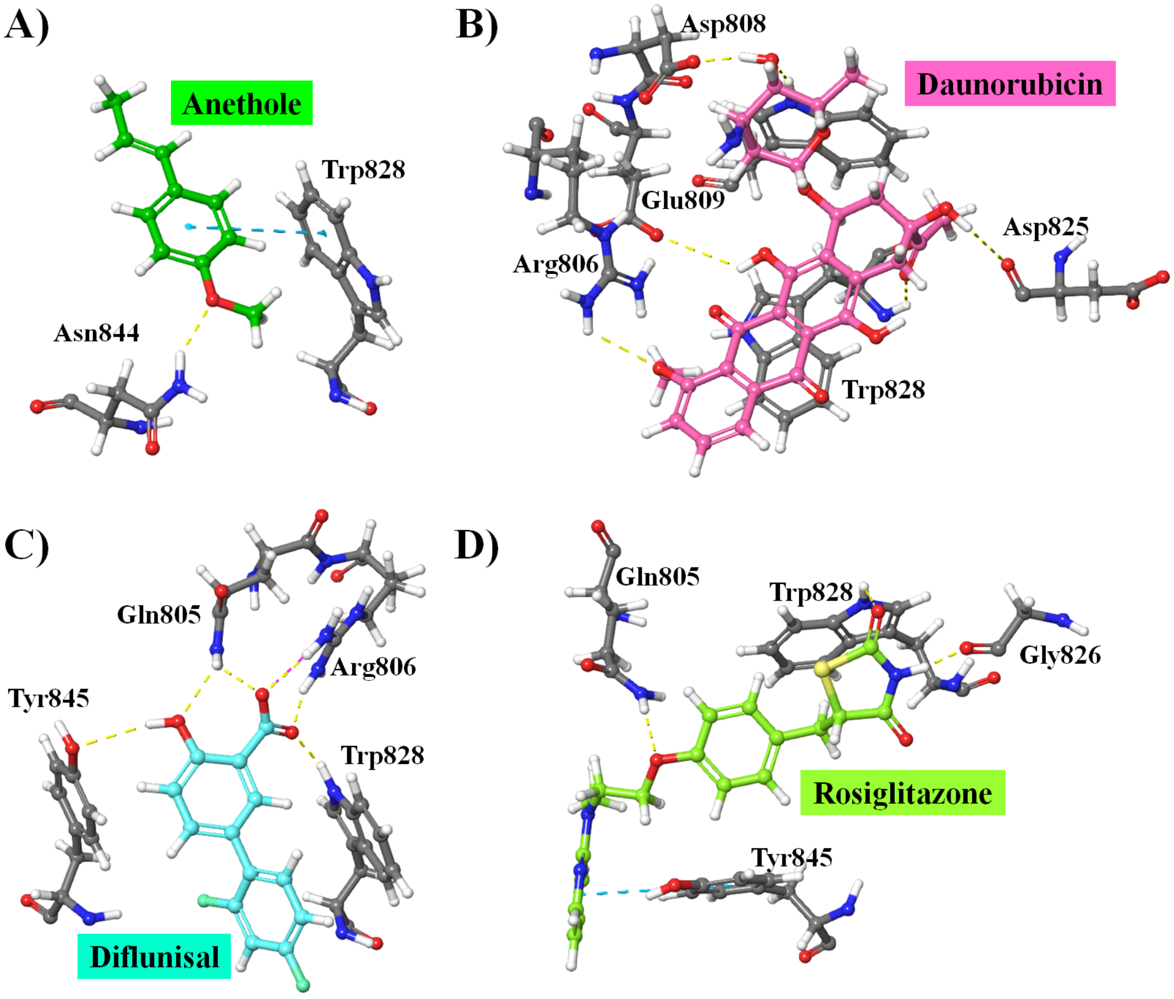

Figure S4. 3D molecular recognition interaction for the reported inhibitors [6] with PLGγ1 after induced fit molecular docking. (A) Anethole. (B) Daunorubicin. (C) Diflunisal. (D) Rosiglitazone. Legend for interactions: hydrogen bonds in yellow; π···cation interactions in green; π···π stacking interactions in blue; aromatic hydrogen bonds in cyan; salt bridges in magenta.

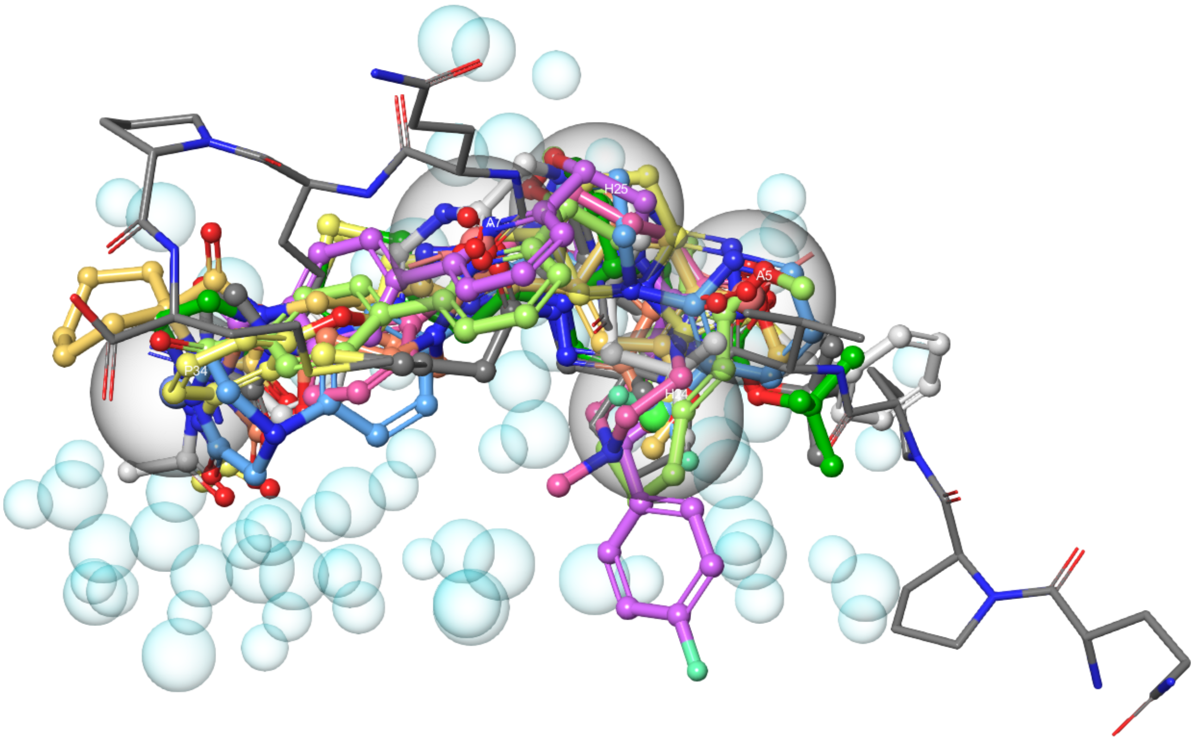

Figure S5. Structural overlap of the top 15 molecules (ball and stick representation) with generated hypothesis and SLP76 (grey sticks).

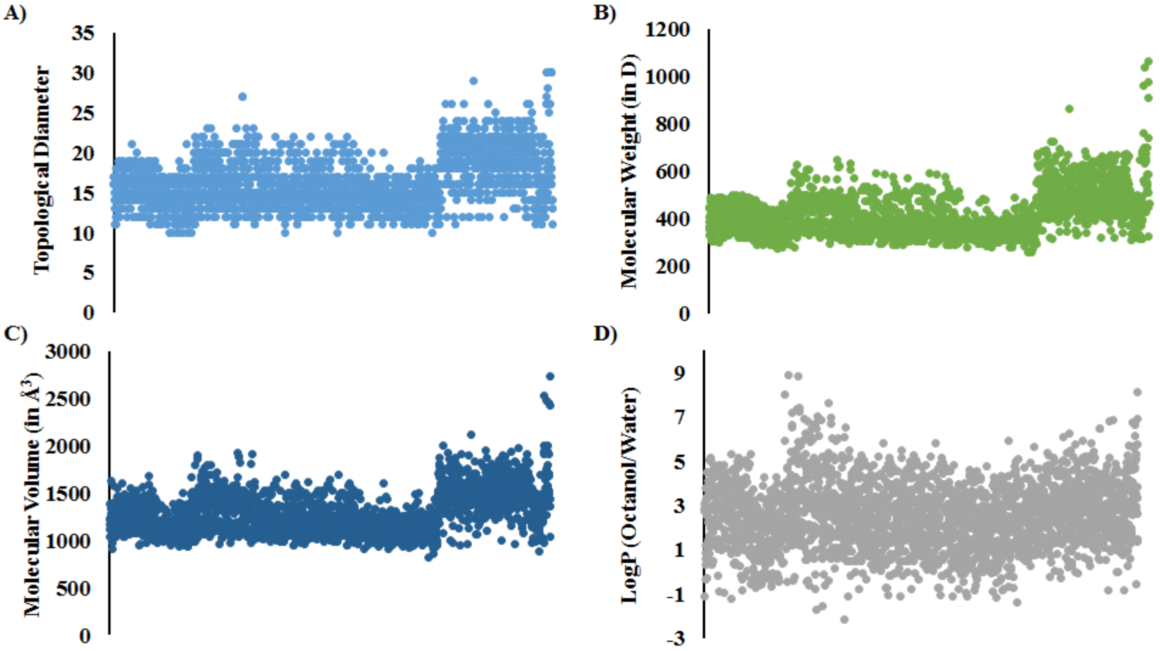

Figure S6. Molecular descriptor analysis for the molecules selected after pharmacophore map based virtual screening. (A) Topological diameter, (B) Molecular weight, (C) Molecular volume and (D) Octanol/water partition coefficient (LogP).

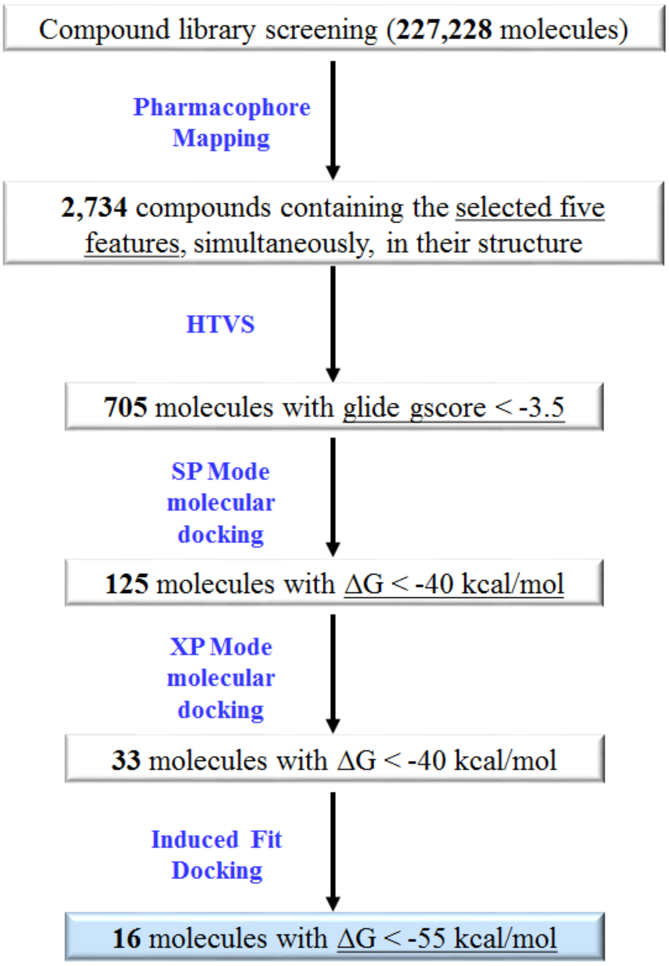

Figure S7. Virtual screening protocol employed in this work.

Table S2. Source library and CAS ID for the 16 molecules, selected after induced fit molecular docking. An internal ID was assigned in this work, so as to facilitate discussion. Formal charge on the ionization state considered for molecular docking are also indicated in the Table.

ID	Library	CAS ID	Charge
IN1	ASINEX	2125345-10-8	1
IN2	ChemDiv	932295-16-4	0
IN3	ChemDiv	434324-08-0	0
IN4	ChemDiv	1029734-12-0	0
IN5	ASINEX	-NA-	2
IN6	ASINEX	2125469-81-8	1
IN7	ChemBridge	1452992-77-6	1
IN8	ChemDiv	434324-08-0	1
IN9	ChemBridge	1351056-16-0	1
IN10	ChemBridge	1360274-41-4	1
IN11	ChemDiv	896697-97-5	0
IN12	ChemBridge	292868-98-5	0
IN13	ChemBridge	1269142-63-3	1
IN14	ChemBridge	1350998-73-0	1
IN15	ChemBridge	1227722-16-8	0
IN16	ChemBridge	1351246-43-9	0

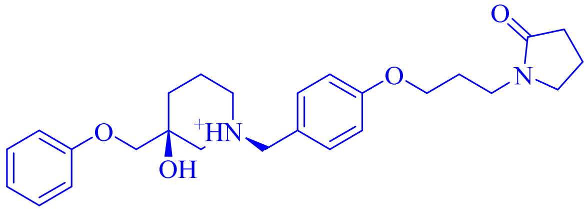

IN1

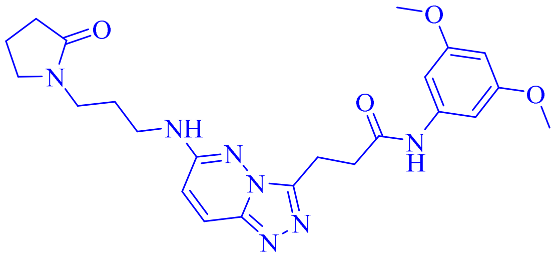

IN2

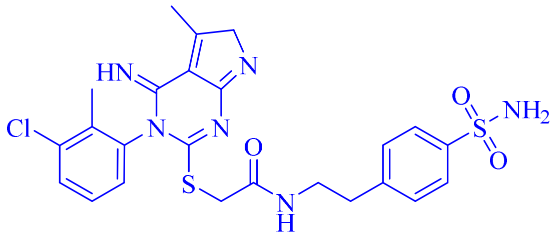

IN3

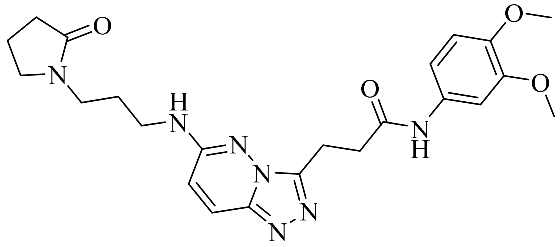

IN4

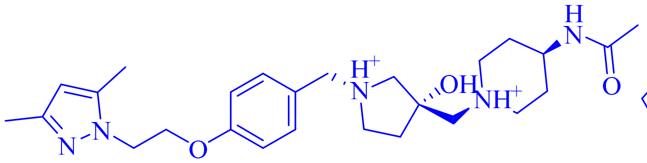

IN5

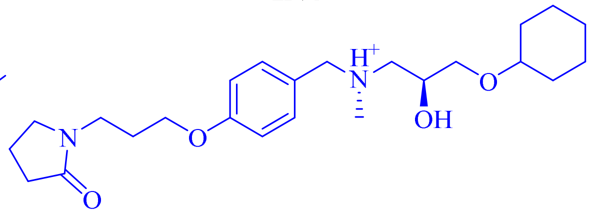

IN6

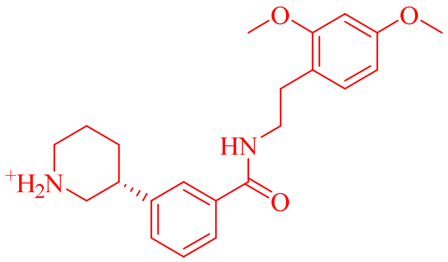

IN7

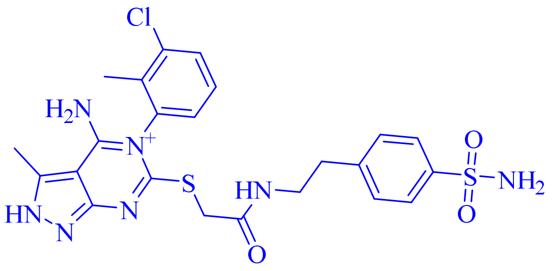

IN8

Figure S8. Structural formula of the molecules IN1 to IN8, selected after molecular docking based virtual screening. Molecules which showed stable binding with PLGγ1 during molecular dynamics (MD) simulations are in blue, whereas molecules which did not exhibited stable binding with PLGγ1 are in red. Molecules shown in black did not exhibit high binding affinity with PLGγ1 during MD simulations.

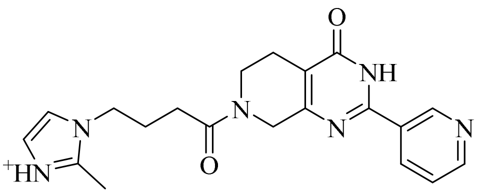

IN9

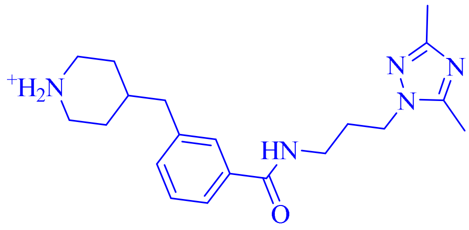

IN10

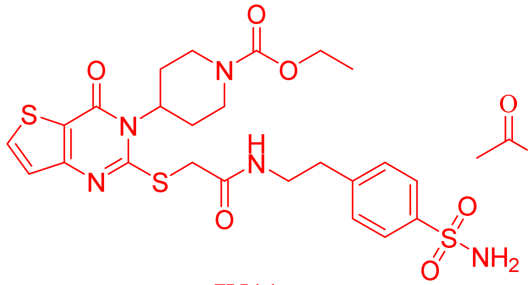

IN11

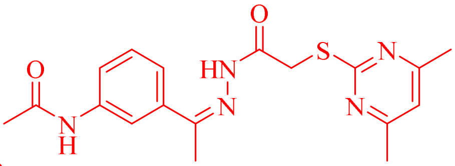

IN12

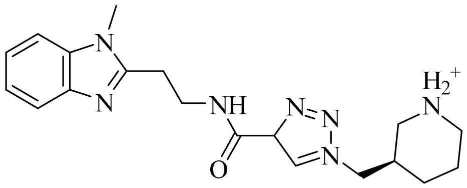

IN13

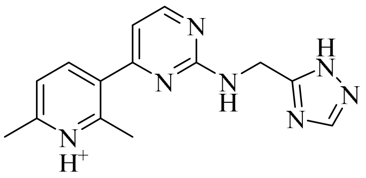

IN14

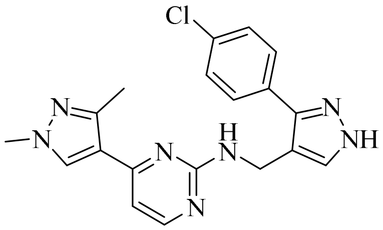

IN15

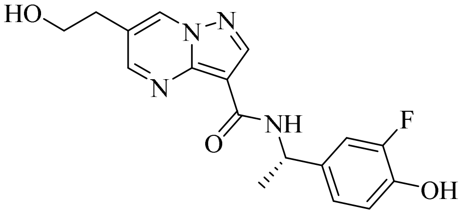

IN16

Figure S9. Structural formula of the molecules IN9 to IN16, selected after molecular docking based virtual screening. Molecules which showed stable binding with PLGγ1 during molecular dynamics simulations are in blue, whereas molecules which did not exhibited stable binding with PLGγ1 are in red. Molecules shown in black did not exhibited high binding affinity with PLGγ1 according to molecular dynamics simulations.

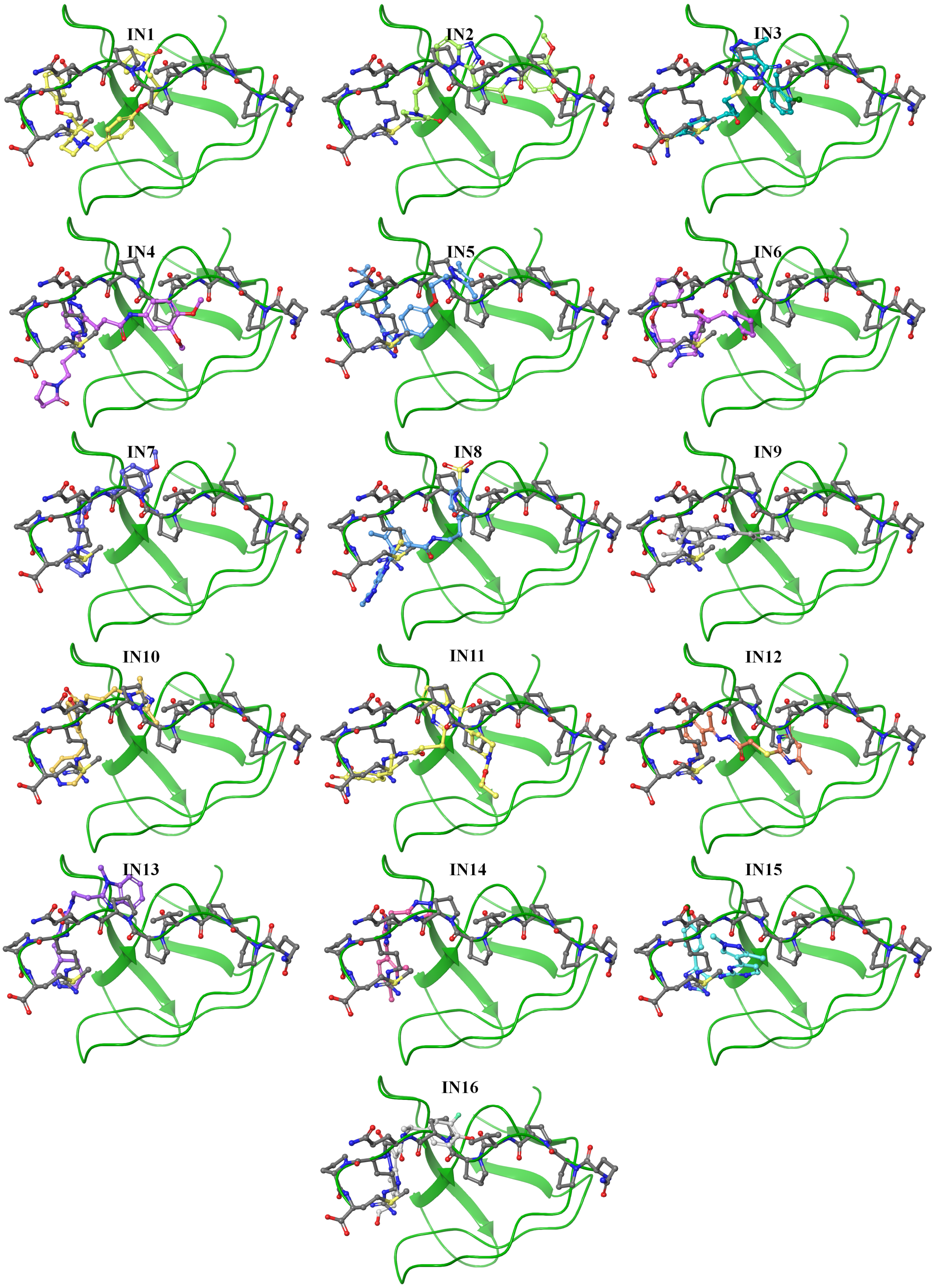

Figure S10. Structural overlap of the 16 molecules IN1-IN16 with SLP76 (in grey) after induced fit molecular docking.

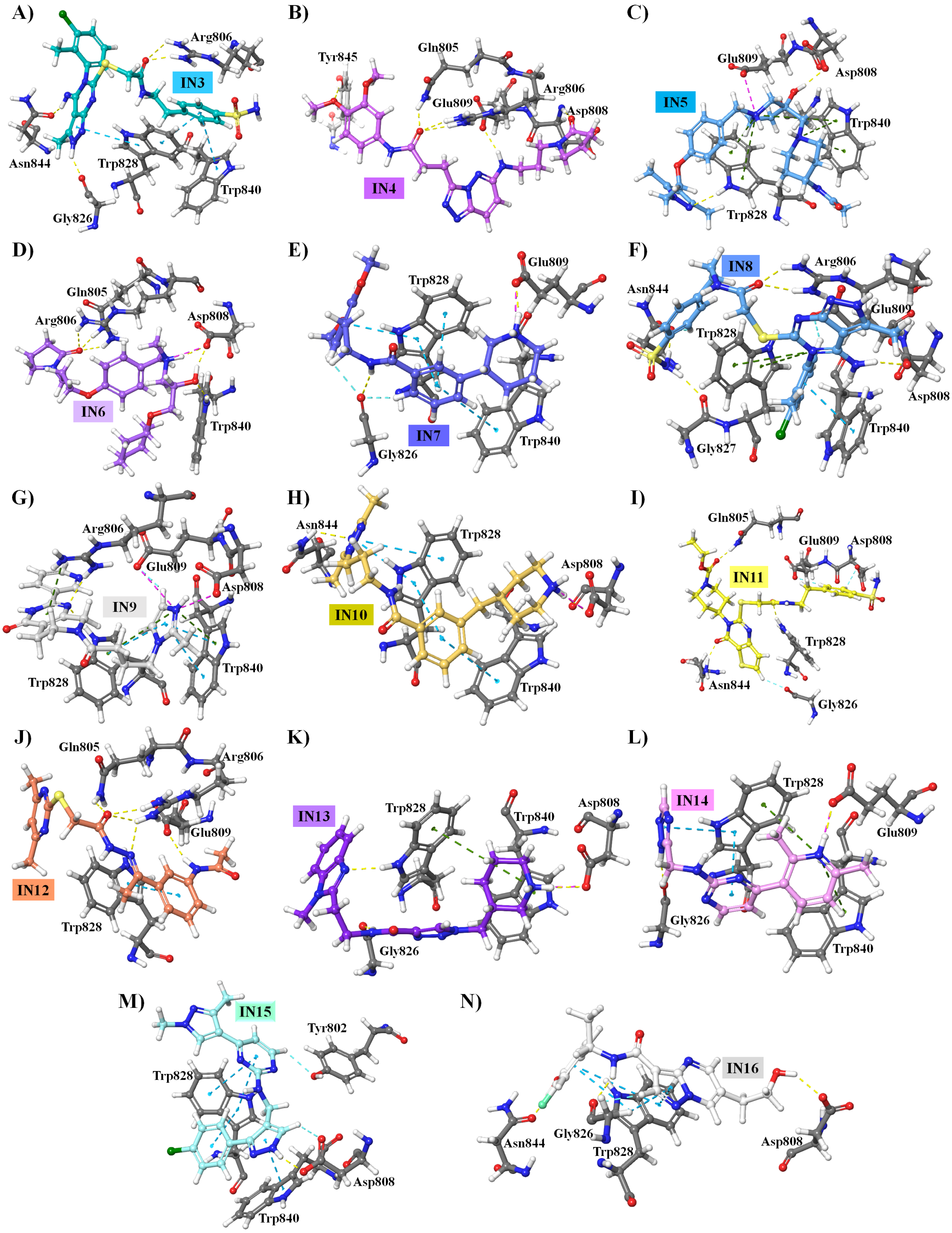

Figure S11. 3D molecular recognition interactions for the various compounds (selected after virtual screening) with PLGγ1 after induced fit molecular docking (see Figure S6 for color legend).

Table S3. MM/GBSA binding energy (ΔG_{bind}) after induced fit molecular docking for the selected 16 ligands.

Molecule ID	Molecular Weight	Molecular Volume	Prime MM/GBSA ΔG_{bind} (kcal/mol)			Weight Normalized $\Delta G_{bind-MW}$ (kcal/mol)	Volume Normalized $\Delta G_{bind-MV}$ (kcal/mol)
			SP Mode	XP Mode	IFD		
Anethole	148.20	613.68	-NA-	-NA-	−35.33	−0.24	−0.06
Daunorubicin	527.53	1459.26	-NA-	-NA-	−38.73	−0.07	−0.03
Diflunisal	250.20	739.34	-NA-	-NA-	−43.55	−0.17	−0.06
Rosiglitazone	357.43	1098.28	-NA-	-NA-	−54.30	−0.15	−0.05
Ritonavir	720.94	2195.15	-NA-	-NA-	−70.12	−0.09	−0.03
SLP76	1146.37	3330.87	-NA-	-NA-	−85.42	−0.07	−0.03
IN1	438.57	1449.53	-57.98	-47.91	−78.07	−0.18	−0.05
IN2	467.53	1466.47	-50.42	-51.01	−75.62	−0.16	−0.05
IN3	546.06	1539.22	-40.99	-43.15	−71.41	−0.13	−0.05
IN4	467.53	1477.27	-45.12	-50.89	−69.51	−0.15	−0.05
IN5	469.63	1609.18	-54.35	-55.42	−68.87	−0.15	−0.04
IN6	418.58	1365.13	-51.17	-46.94	−66.04	−0.16	−0.05
IN7	368.48	1264.40	-47.38	-51.25	−63.57	−0.17	−0.05
IN8	546.06	1539.22	-47.38	-58.05	−62.99	−0.11	−0.04
IN9	378.43	1218.03	-49.92	-52.18	−62.74	−0.17	−0.05
IN10	355.48	1276.66	-42.63	-50.03	−62.63	−0.18	−0.05
IN11	579.70	1657.15	-45.28	-53.49	−62.62	−0.11	−0.04
IN12	371.46	1254.61	-46.65	-46.84	−61.29	−0.16	−0.05
IN13	367.45	1238.62	-47.43	-46.48	−59.28	−0.16	−0.05
IN14	281.32	917.52	-44.37	-44.55	−59.16	−0.21	−0.06
IN15	379.85	1174.97	-51.61	-51.51	−58.92	−0.15	−0.05
IN16	344.34	1075.63	-40.88	-45.97	−56.34	−0.16	−0.05

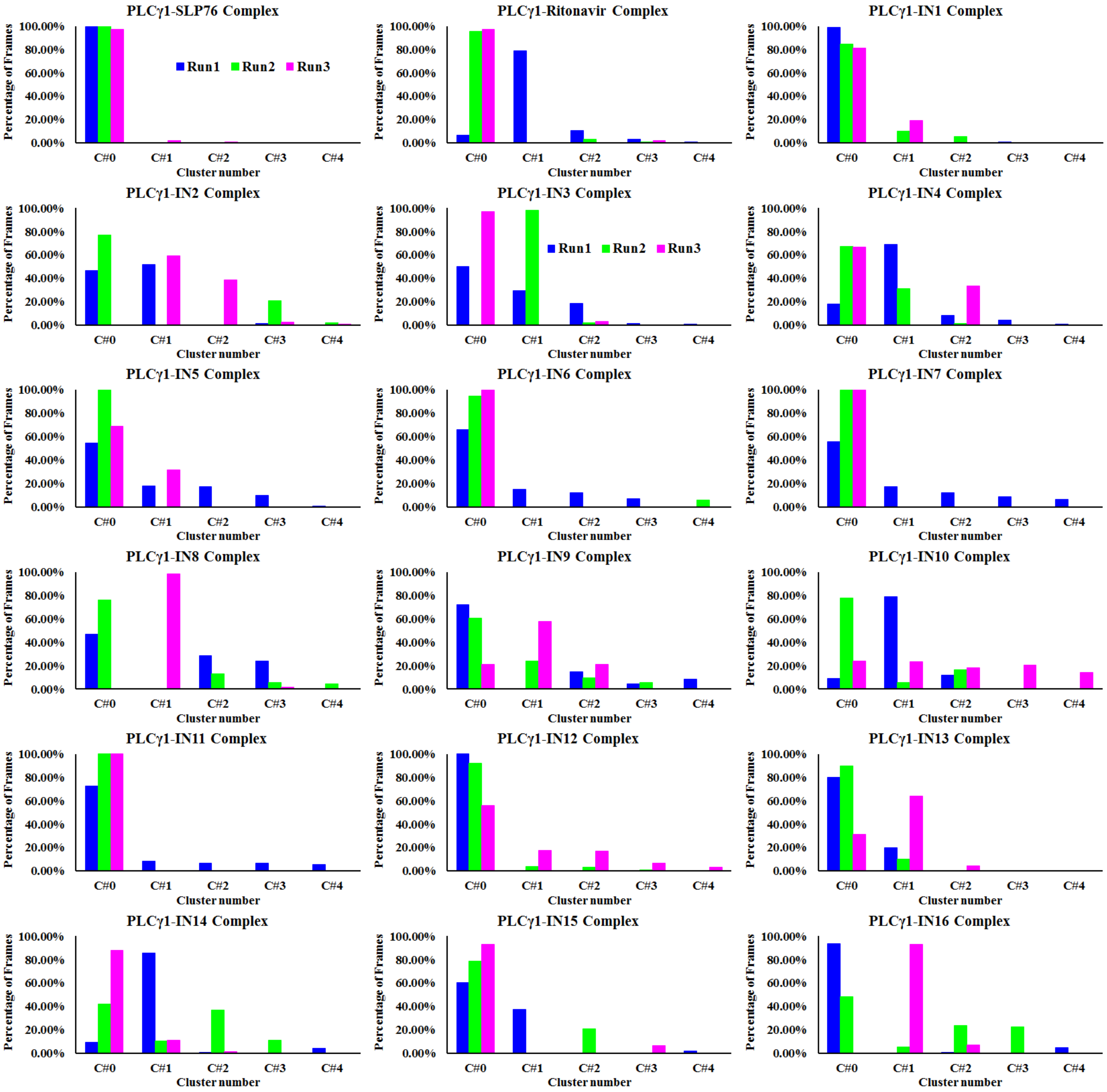

Figure S12. Combined clustering analysis to evaluate the reproducibility of the MD simulations in three replicates.

Table S4. RMSD between the clusters generated from the molecular dynamics simulation trajectories with combined clustering analysis of three replicate runs.

Title	RMSD from C#0 (Å)			
	C#1	C#2	C#3	C#4
SLP76	2.30	2.39	2.05	1.71
Ritonavir	1.92	1.57	1.59	1.99
IN1	1.85	1.91	1.91	1.54
IN2	1.95	1.61	1.60	1.60
IN3	1.80	1.94	1.82	1.69
IN4	1.62	1.75	1.67	2.05
IN5	1.60	1.76	1.49	2.06
IN6	1.58	1.83	1.46	1.87
IN7	2.16	2.03	2.05	2.19
IN8	1.85	1.52	1.53	1.98
IN9	1.63	1.54	1.54	1.43
IN10	1.62	1.58	1.75	1.82
IN11	1.84	1.59	1.72	1.76
IN12	1.75	1.60	1.64	1.80
IN13	1.42	1.78	1.73	1.77
IN14	1.72	1.57	1.69	2.34
IN15	1.66	1.67	1.85	1.79
IN16	1.63	1.48	1.82	1.69

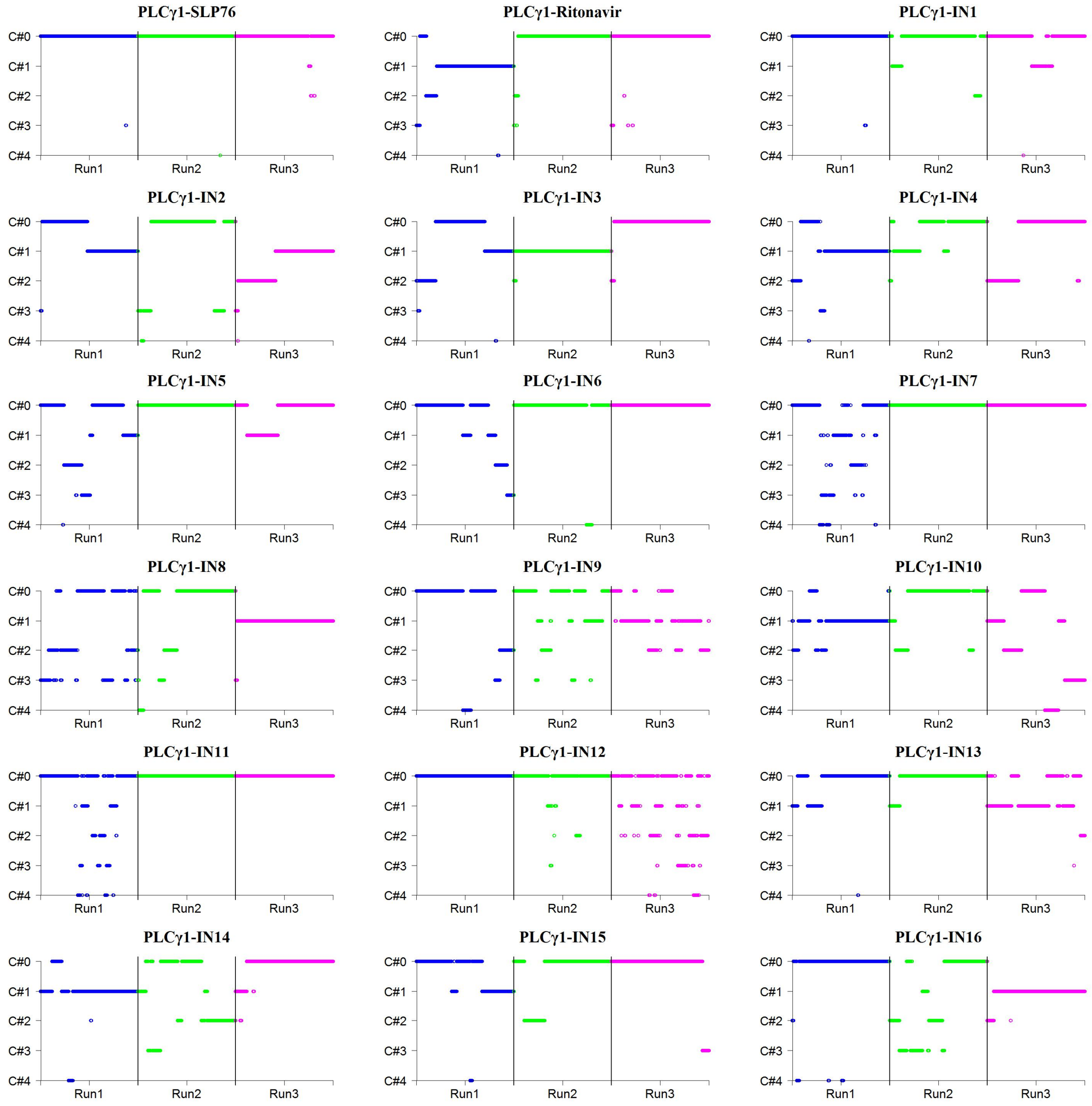

Figure S13. Cluster population *vs.* time for the three replicates molecular dynamics simulations of the eighteen complexes systems. Y-axis represents the cluster number.

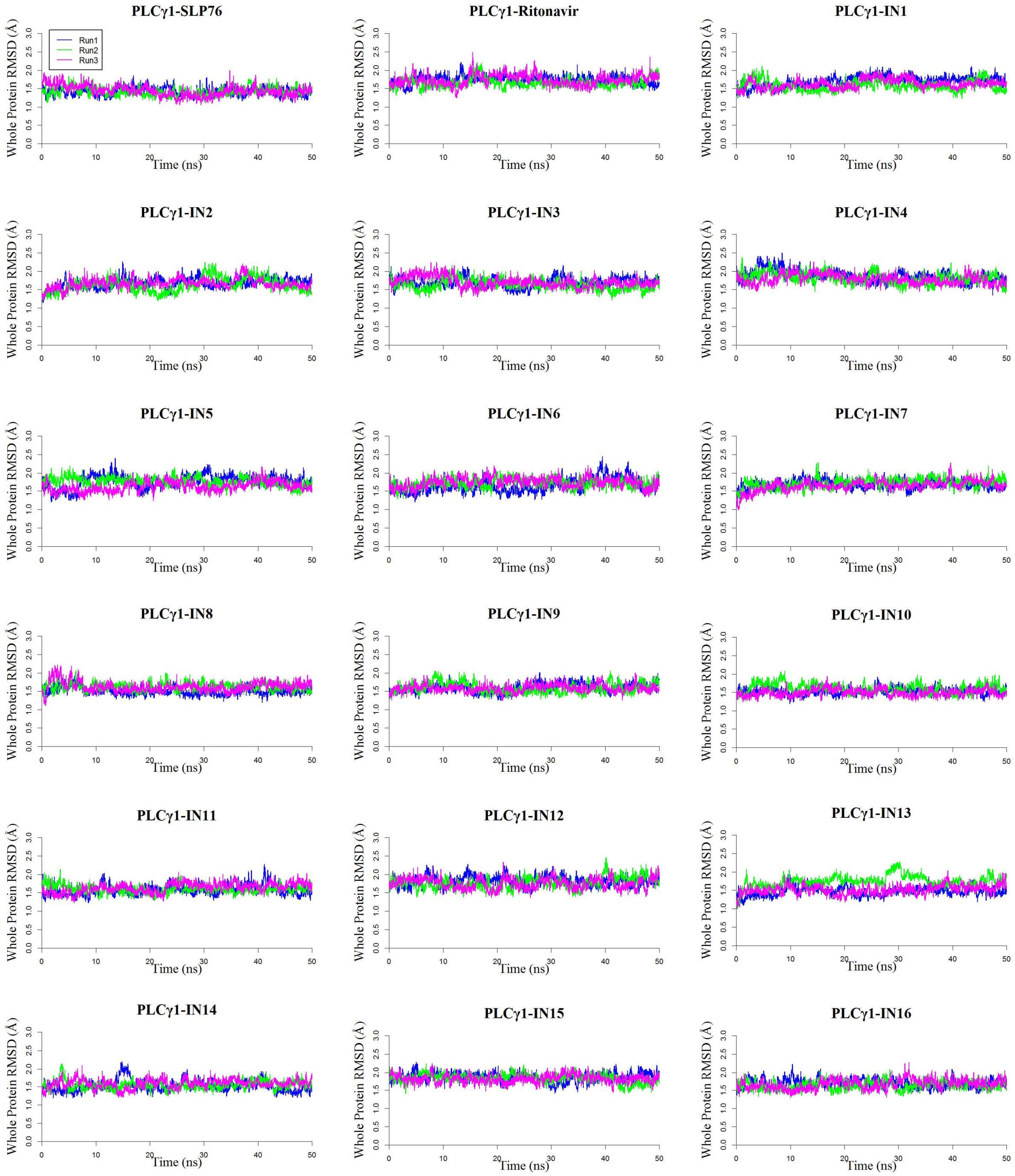

Figure S14. Whole protein RMSD for PLGγ1 in the eighteen complexes from the three replicate molecular dynamics simulations.

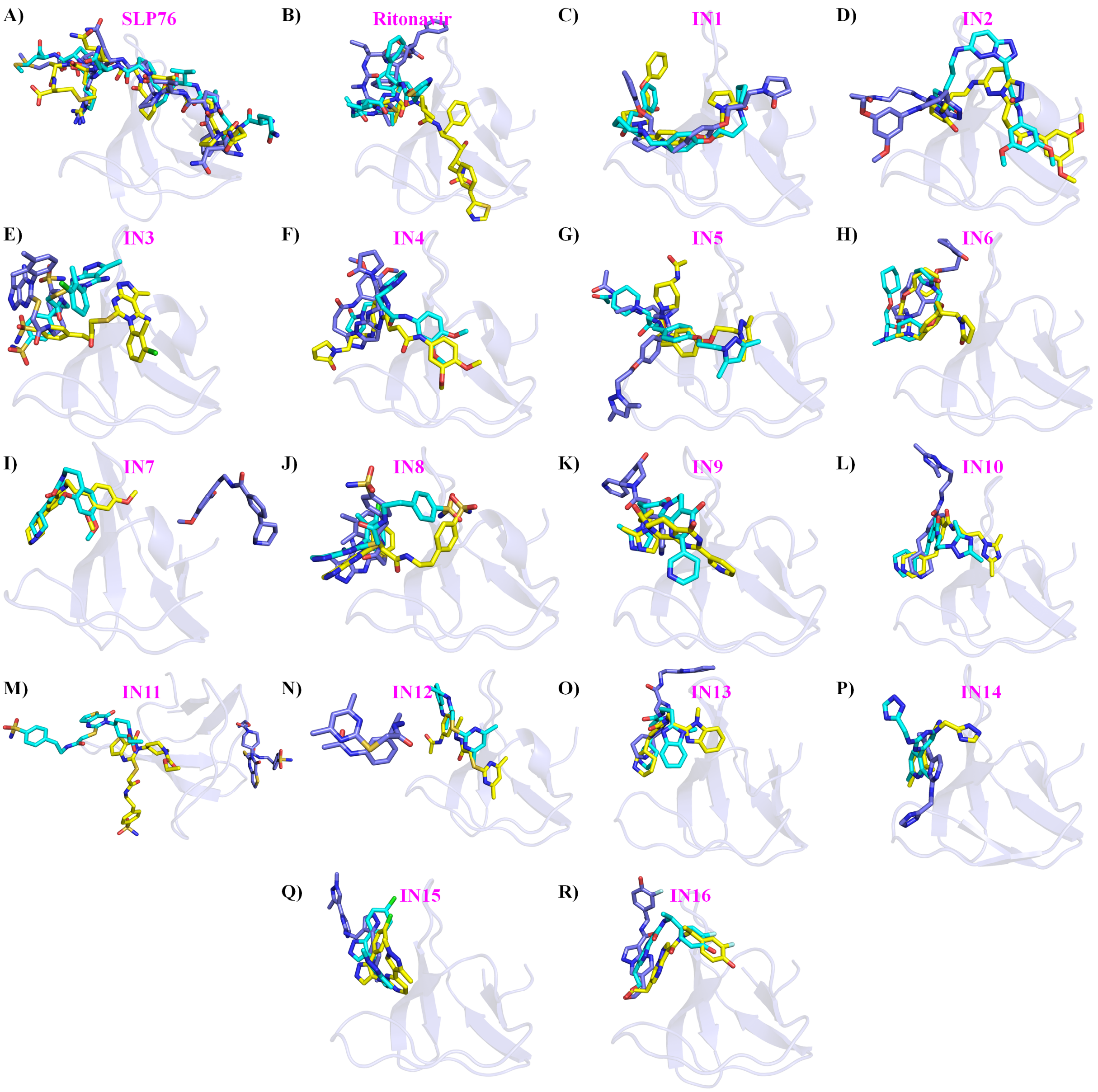

Figure S15. Structural overlap for the eighteen complexes generated after molecular docking (yellow), equilibration (cyan) and molecular dynamics simulation (blue).

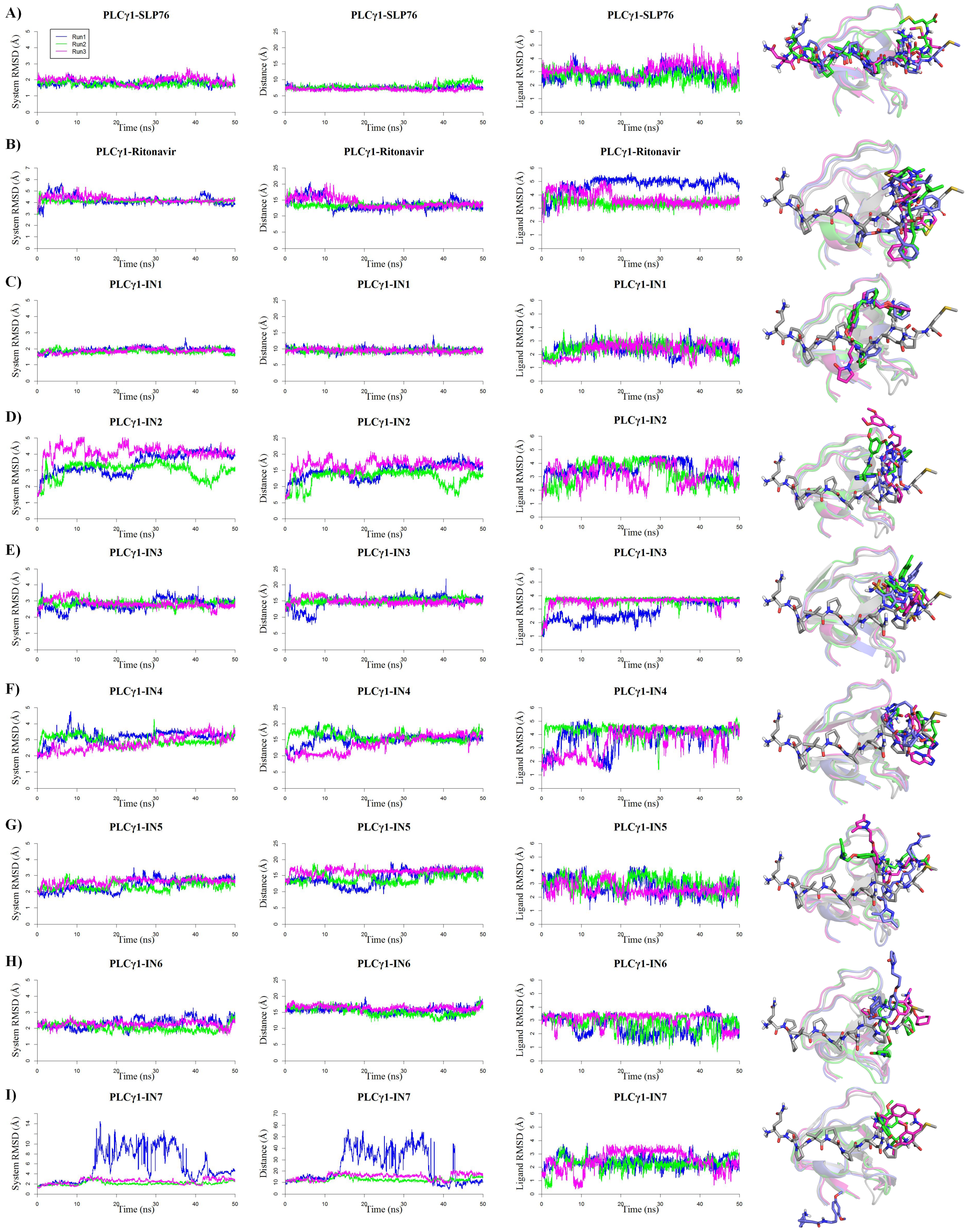

Figure S16. Whole system RMSD, distance between the center of mass of the bound ligand and AsnA844 of PLGγ1, ligand RMSD, and structure overlap of the last coordinates from the three replicate molecular dynamics simulations of the complexes containing SLP76, ritonavir and identified hits (IN1 to IN7).

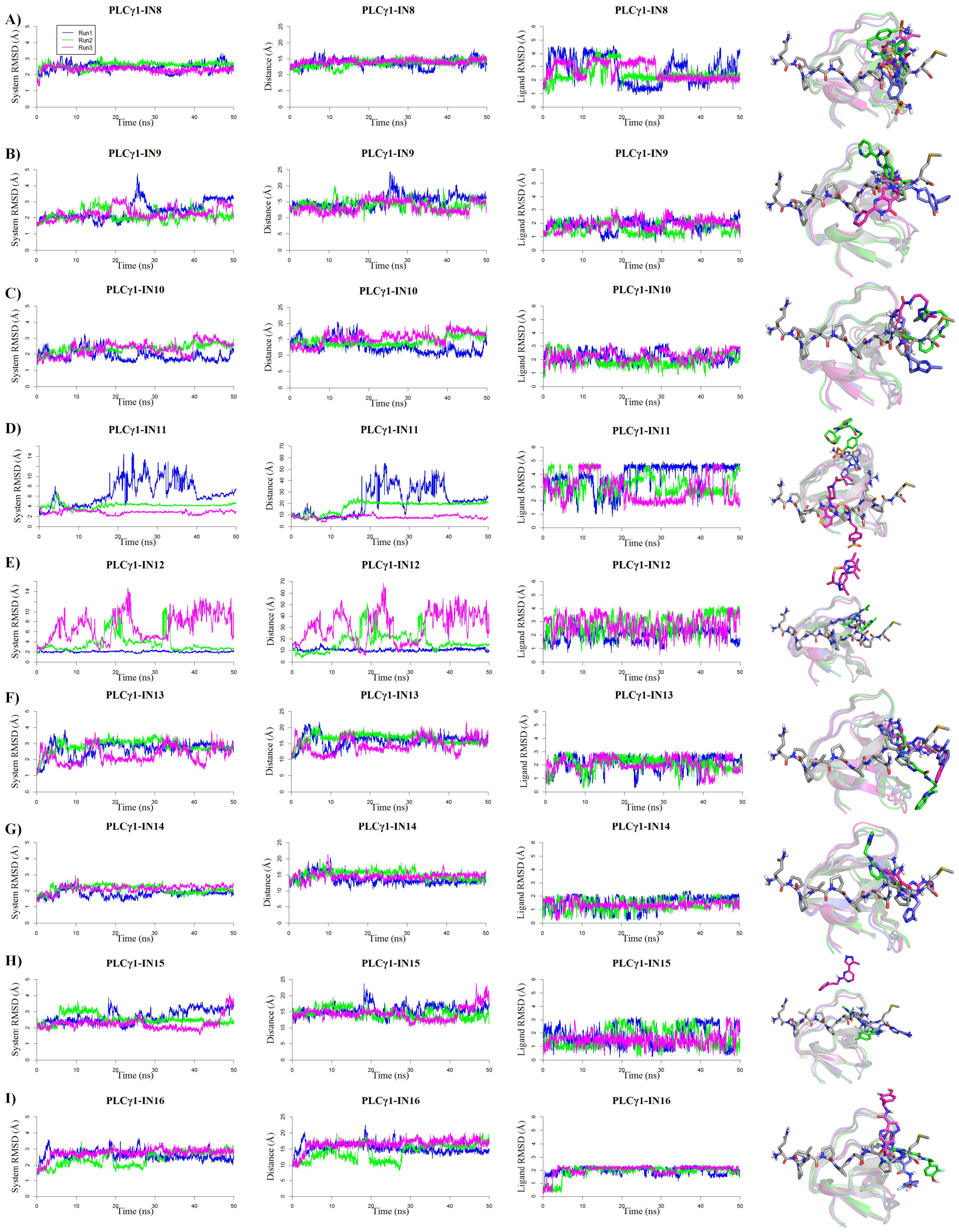

Figure S17. Whole system RMSD, distance between center of mass of the bound ligand and AsnA844 of PLGγ1, ligand RMSD, and structure overlap of the last coordinates from the three replicate of molecular dynamics simulations of the complexes containing identified hits (IN8 to IN16).

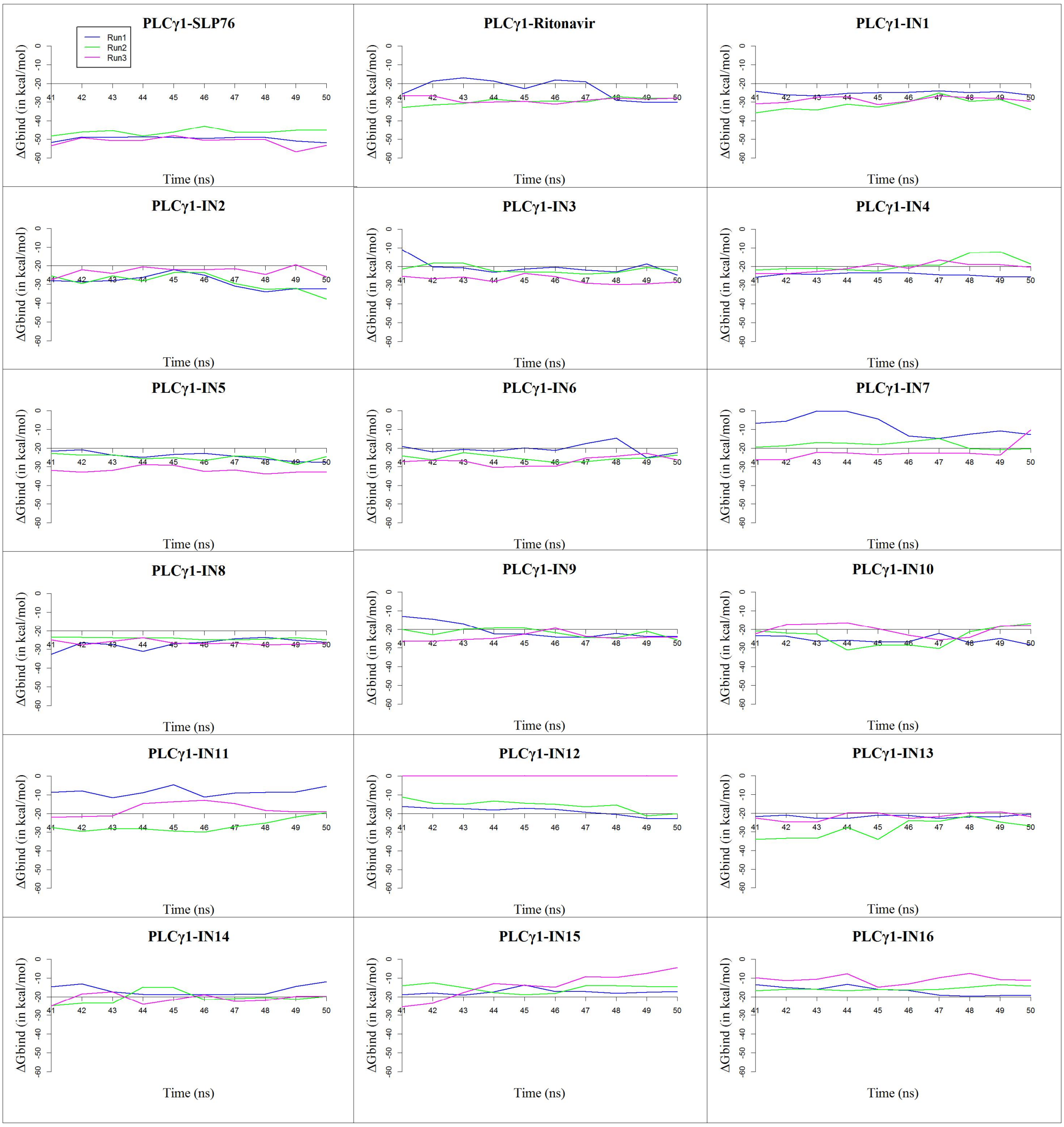

Figure S18. Per-nanosecond MM-GBSA binding energy (ΔG_{bind}) calculated for sixteen PLGγ1 complexes obtained from the molecular dynamics (MD) simulations. Results are shown only for the ligands, showing stable binding during the three replicate MD simulations.

Table S5. Average binding energy results (calculated over last 5 ns using MM-GBSA method) for complexation of various ligands with PLCγ1 along with the different energy components.[a]

Title	vdW	EEL	EGB	ESURF	ΔG_{gas}	ΔG_{solv}	$\Delta G_{bind} \pm$ SD*	SE*
**SLP76**	−43.92	−119.92	119.79	−6.09	−163.84	113.70	−50.14 ± 3.96	0.25
**Ritonavir**	−35.84	−15.33	26.77	−4.35	−51.17	22.42	−28.75 ± 2.76	0.17
**IN1**	−27.23	−92.84	95.94	−4.16	−120.07	91.78	−28.29 ± 3.05	0.19
**IN2**	−36.19	−26.51	35.53	−4.15	−62.70	31.38	−31.32 ± 5.62	0.36
**IN3**	−27.66	−25.19	28.09	−3.64	−52.85	24.45	−28.40 ± 3.92	0.25
**IN4**	−33.48	−9.43	21.51	−3.36	−42.91	18.15	−24.76 ± 2.87	0.18
**IN5**	−30.39	−163.42	164.97	−3.83	−193.81	161.14	−32.67 ± 3.33	0.21
**IN6**	−23.67	−114.31	115.58	−3.49	−137.98	112.09	−25.89 ± 3.42	0.22
**IN7**	−13.07	−124.91	127.50	−2.40	−137.98	125.10	−12.88 ± 4.23	0.27
**IN8**	−34.33	−95.91	106.22	−3.03	−130.24	103.19	−27.05 ± 2.49	0.16
**IN9**	−29.02	−85.89	94.91	−3.73	−114.91	91.18	−23.71 ± 3.42	0.22
**IN10**	−21.89	−100.66	99.64	−2.93	−122.55	96.71	−25.84 ± 3.42	0.22
**IN11**	−14.62	−2.14	9.78	−1.63	−16.76	8.15	−8.61 ± 3.09	0.19
**IN12**	−0.03	−0.04	0.14	0.00	−0.07	0.14	0.07 ± 0.13	0.01
**IN13**	−28.51	−93.39	100.83	−3.19	−121.90	97.64	−24.26 ± 3.76	0.24
**IN14**	−20.25	−76.12	78.15	−2.66	−96.37	75.49	−20.88 ± 3.02	0.19
**IN15**	−25.23	−8.61	19.35	−2.92	−33.84	16.43	−17.41 ± 2.62	0.16
**IN16**	−28.55	−19.56	32.78	−3.40	−48.11	29.38	−18.73 ± 2.72	0.17

[a] The meaning of the different terms used in this table is as follows: VDW = van der Waals energy as calculated by the MM force field. EEL = electrostatic energy as calculated by the MM force field. EGB = the electrostatic contribution to the solvation free energy calculated by GB. ESURF = nonpolar contribution to the solvation free energy calculated by an empirical model. ΔGgas = total gas phase energy i.e. sum of van der Waals and electrostatic energy from MM. ΔGsolv = total solvation free energy i.e. sum of electrostatic and nonpolar contributions from solvation. ΔGbind = final estimated binding free energy calculated from the terms above (kcal/mol).

*SD: Standard Deviation; SE: Standard Error of Mean

Table S6. Weight-based and volume based normalization of average binding free energy for complexation of various ligands with PLCγ1.

Enzyme	Molecular Weight	Molecular Volume	ΔG_{bind} (kcal/mol)	Weight Normalized $\Delta G_{bind-MW}$ (kcal/mol)	Volume Normalized $\Delta G_{bind-MV}$ (kcal/mol)
**SLP76**	1146.369	3330.865	−50.145	−0.044	−0.015
**Ritonavir**	720.943	2195.149	−28.754	−0.040	−0.013
**IN1**	438.566	1449.535	−28.294	−0.065	−0.020
**IN2**	467.527	1466.466	−31.321	−0.067	−0.021
**IN3**	546.061	1539.222	−28.400	−0.052	−0.018
**IN4**	467.527	1477.274	−24.764	−0.053	−0.017
**IN5**	469.626	1609.179	−32.670	−0.070	−0.020
**IN6**	418.575	1365.129	−25.899	−0.062	−0.019
**IN7**	368.475	1264.400	−12.885	−0.035	−0.010
**IN8**	546.061	1539.222	−27.057	−0.050	−0.018
**IN9**	378.433	1218.031	−23.708	−0.063	−0.019
**IN10**	355.482	1276.655	−25.842	−0.073	−0.020
**IN11**	579.703	1657.149	−8.606	−0.015	−0.005
**IN12**	371.456	1254.612	0.072	0.000	0.000
**IN13**	367.453	1238.625	−24.261	−0.066	−0.020
**IN14**	281.319	917.520	−20.884	−0.074	−0.023
**IN15**	379.851	1174.969	−17.411	−0.046	−0.015
**IN16**	344.345	1075.633	−18.729	−0.054	−0.017

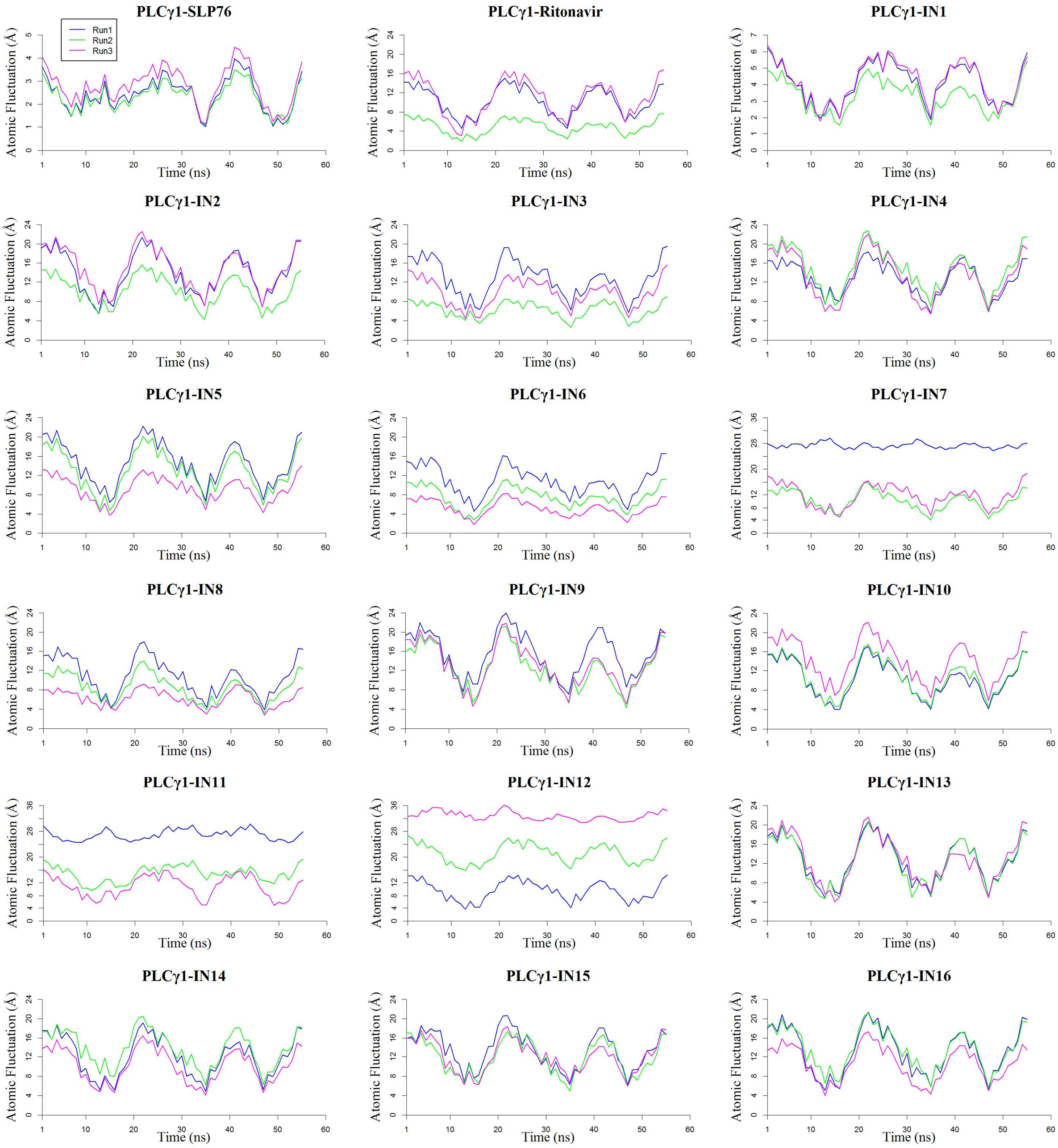

Figure S19. Per residue atomic fluctuation analysis for PLGγ1 in eighteen complexes considered from the three replicate molecular dynamics simulations.

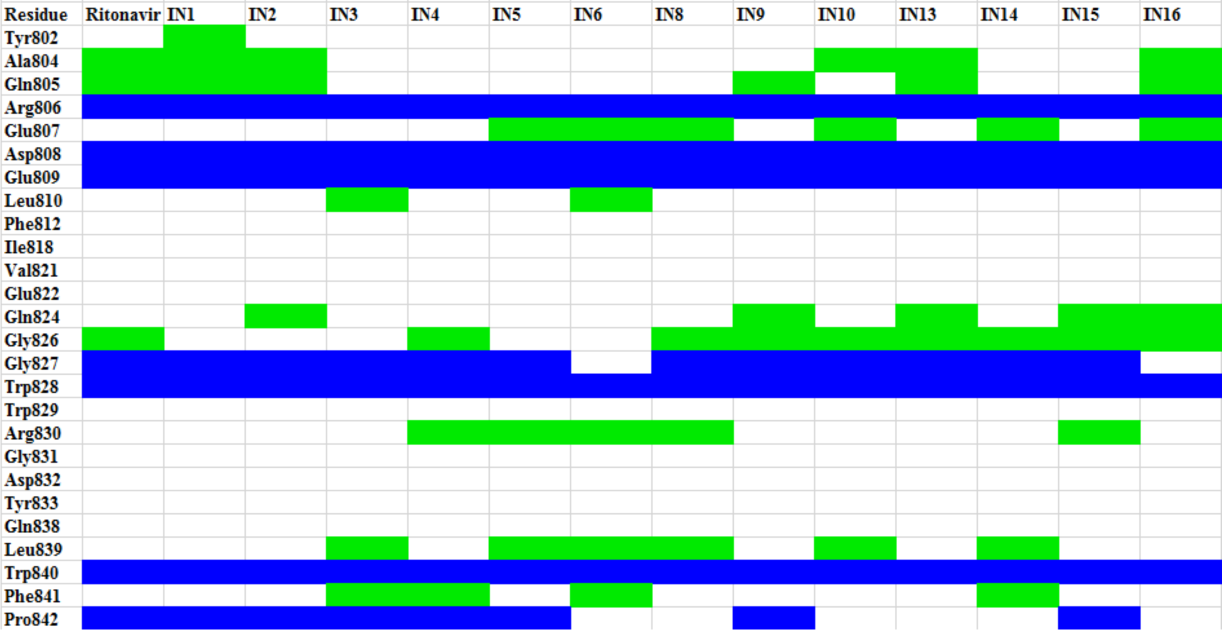

Figure S20. PLGγ1 residues which showed lower atomic fluctuation during molecular dynamics simulations in sixteen complexes. Cells highlighted in green indicate that the residue was in the top 10 residues with lower fluctuation; cells in blue indicate the residues which showed lower fluctuation in majority of systems, and thus can be important in complex formation.

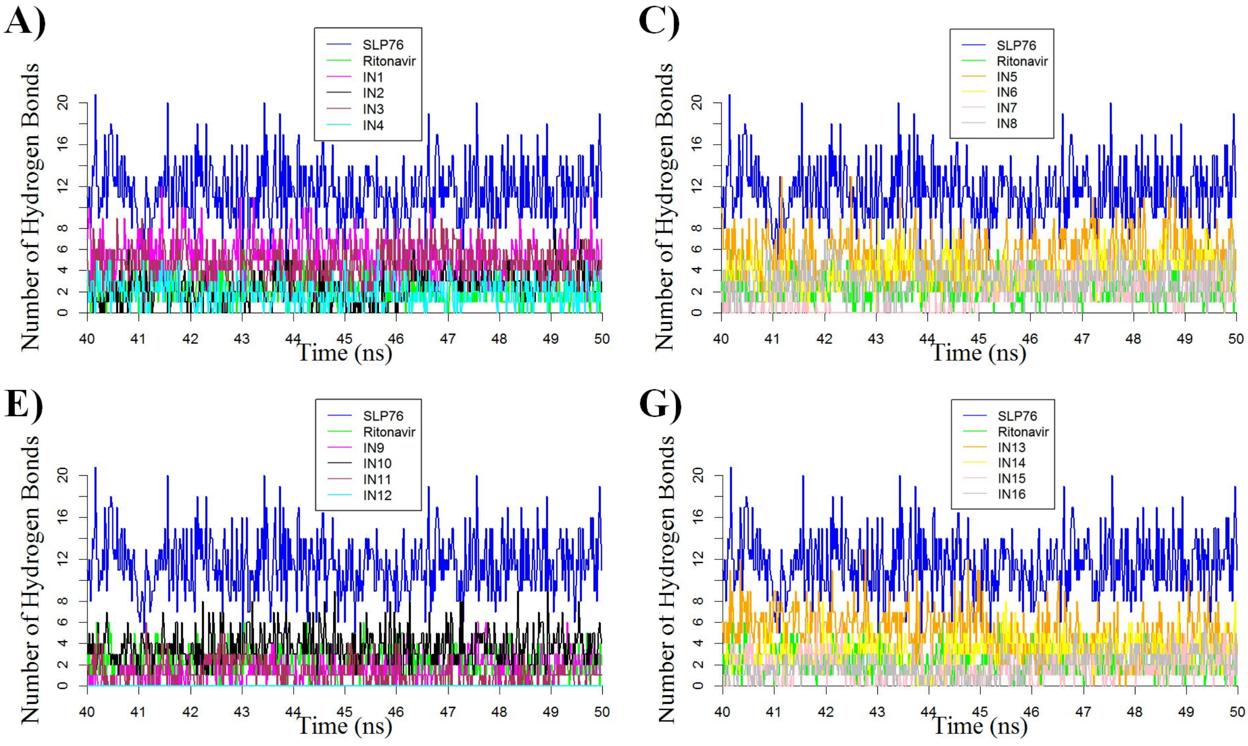

Figure S21. Number of hydrogen bonds over the last 10 ns between ligand and PLCγ1 in various complexes from the three replicate molecular dynamics.

Table S7. Average number of hydrogen bonds between the ligand and PLCγ1 over the last 10 ns simulation run. Ligands with more than 3 hydrogen bonds with PLCγ1 are highlighted in green.

Title	Average number of hydrogen bonds
SLP76	12
RIT	2
IN1	5
IN2	2
IN3	5
IN4	2
IN5	5
IN6	4
IN7	1
IN8	3
IN9	2
IN10	4
IN11	1
IN12	0
IN13	5
IN14	3
IN15	2
IN16	2

Table S8. Molecules considered for virtual screening.

Sl. No.	Library	Number of compounds
1	ASINEX	11,377
2	Chembridge	1,00,000
3	Chemdiv	12,995
4	Enamine	65,512
5	LifeChemicals	22,944
6	Maybridge	14,400
	**Total**	**227,228**

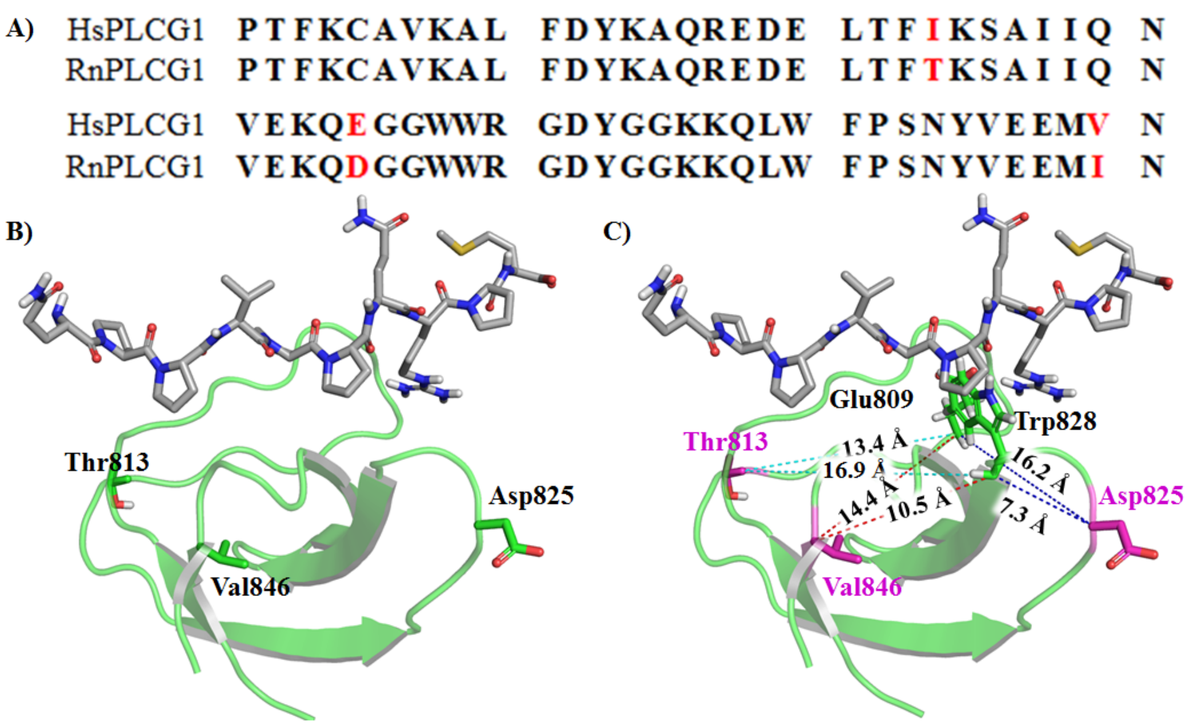

Figure S22. Comparative analysis of the PLCγ1-SH3 domain from Rattus norvegicus (UniProt ID: P10686) and Homo sapiens (UniProt ID: P19174). (A) Sequence alignment to identify non-identical residues (in red) between the two sequences. (B) Topographical location of identified residues (stick representation in green) in the crystal structure of PLCγ1-SLP76 complex from Rattus norvegicus (PDB ID: 1YWO) [5]. (C) Distance analysis of the non-identical residues (sticks representation in magenta) from the arginine binding site (Glu809) and proline recognition site (Trp828). Legend for colors: cyan for Thr813, blue for Asp825 and red for Val846.

References

1. Yang, Y.R.; Choi, J.H.; Chang, J.-S.; Kwon, H.M.; Jang, H.-J.; Ryu, S.H.; Suh, P.-G. Diverse cellular and physiological roles of phospholipase C-γ1. *Adv. Enzyme Regul.* **2012**, *52*, 138–151.
2. Koss, H.; Bunney, T.D.; Behjati, S.; Katan, M. Dysfunction of phospholipase Cγ in immune disorders and cancer. *Trends Biochem. Sci.* **2014**, *39*, 603–611.
3. Kadamur, G.; Ross, E.M. Mammalian Phospholipase C. *Annu. Rev. Physiol.* **2012**, *75*, 127–154.
4. Gierschik, P.; Buehler, A.; Walliser, C. Activated PLCγ breaking loose. *Structure* **2012**, *20*, 1989–1990.
5. Deng, L.; Velikovsky, C.A.; Swaminathan, C.P.; Cho, S.; Mariuzza, R.A.; Huber, R. Structural basis for recognition of the T Cell adaptor protein SLP-76 by the SH3 domain of phospholipase Cγ1. *J. Mol. Biol.* **2005**, *352*, 1–10.
6. Poissonnier, A.; Guégan, J.P.; Nguyen, H.T.; Best, D.; Levoin, N.; Kozlov, G.; Gehring, K.; Pineau, R.; Jouan, F.; Morere, L.; et al. Disrupting the CD95–PLCγ1 interaction prevents Th17-driven inflammation. *Nat. Chem. Biol.* **2018**, *14*, 1079–1089.
